# Zaraa Uul: An archaeological record of Pleistocene-Holocene palaeoecology in the Gobi Desert

**DOI:** 10.1371/journal.pone.0249848

**Published:** 2021-04-08

**Authors:** Lisa Janz, Arlene M. Rosen, Dashzeveg Bukhchuluun, Davaakhuu Odsuren

**Affiliations:** 1 Department of Anthropology, Trent University, Peterborough, Ontario, Canada; 2 School of Anthropology, University of Arizona, Tucson, Arizona, United States of America; 3 Department of Anthropology, University of Texas at Austin, Austin, Texas, United States of America; 4 Institute of Archaeology, Mongolian Academy of Science, Ulaanbaatar, Mongolia; 5 Department of Anthropology, Yale University, New Haven, Connecticut, United States of America; Max Planck Institute for the Science of Human History, GERMANY

## Abstract

Environmentally-based archaeological research at Zaraa Uul, including zooarchaeology, phytolith analysis, and radiocarbon dating, is the first of its kind in Mongolia and presents critical new insight on the relationship between periods of occupational intensity and climatic amelioration from the earliest anatomically modern humans to the adoption of pastoralism. The palaeoenvironmental and faunal record of Zaraa Uul show that Early-Middle Holocene hydrology and species distributions were distinct from all other periods of human occupation. Holocene hunter-gatherers inhabited an ecosystem characterized by extensive marshes, riparian shrub and arboreal vegetation along the hill slopes and drainages. The exploitation of species associated with riparian and wetland settings supports the hypothesis of, but suggests an earlier timing for, oasis-based logistical foraging during the Early-Middle Holocene of arid Northeast Asia. The onset of wetter conditions at 8500 cal BP agrees with other regional studies, but multiple lines of evidence present the first integrated field- and laboratory-based record of human-environment relationships in arid East Asia during the Holocene Climatic Optimum. We compare it to Late Pleistocene climatic amelioration, and highlight specific responses of the hydrological, vegetative and faunal communities to climate change in arid Northeast Asia.

## Introduction

Wetlands are a critical resource for hunter-gatherers in many environmental contexts [1] and evidence for intensified use of wetlands is especially well represented globally during the Late Pleistocene and early to Middle Holocene [2–9]. Janz [10] posits that the global scale of this trend is deeply embedded in ecological state shifts that drove broadly contemporaneous large-scale changes in decreased residential mobility, broader spectrum diets, heightened reproduction, and more complex social organization. The intensified use of wetlands was a critical component of these changes in many regions, including the Gobi Desert of Mongolia and China. Despite a lack of direct data on diet, massive organizational change is attested during the Middle Holocene, including within both the structure of lithic reduction strategies and the unique spatial patterning of habitation sites [10, 11]. The preferential use of wetlands is clear; however, it is poorly understood how wetlands were used because of a lack of direct data on diet. Janz [10] has suggested that the underlying pattern in land-use is tied directly to increased diet breadth and an emphasis on *r*-selected prey beginning at 8000 cal BP. Her proposed chronology for the region is summarized in Table 1.

**Table 1:** Summary of chronological phases and associated technologies.

Chronological Phases	Dates	Organizational Strategy	Tool Types
**Oasis 1 (Mesolithic)**	13.5–8.0 k cal yr BP	High residential mobility, use of wetlands	Microblade technology; expedient core and flake; pottery by 9600 cal BP
**Oasis 2 (Early Neolithic)**	8.0–5.0 k cal yr BP	Reduced residential mobility, wetland-centric logistical foraging	Microblade technology; more formal flake cores; large formal milling stones; chipped and/or polished adzes and axes; bifacial projectile points; textile-impressed pottery
**Oasis 3 (Eneolithic, Bronze Age)**	5.0–3.0 k cal yr BP	Wetland-centric logistical foraging, introduction of domesticated herd animals	Microblade technology; increased in use of flake cores; bifacially-flaked arrowheads, blades, knives; milling stones; copper slag; increased use of local lithic materials; moulded rim coarse redware, geometric incised

This chronology, beginning with the earliest verified sites post-dating the Last Glacial Maximum (LGM) and continuing into the early stages of herding and bronze metallurgy, is the only date-based chronology for the region and is grounded on site assemblages collected by major interdisciplinary scientific expeditions during the 1920s and 1930s [11–13]. While the use of existing collections has been productive in building a preliminary framework for understanding the relationship between human and landscape palaeoecology, testing requires on-site analysis of both archaeological and geomorphological contexts. Zaraa Uul was discovered in 2013 and excavations during 2015–2018 have produced the first comprehensive faunal assemblage for the Gobi Desert region, including components dating to late MIS 3 and early MIS 1. Here, we present our findings of zooarchaeological and geomorphological analysis and situate those findings within the context of specific ecological and organizational trends in arid East Asia during the Middle Holocene and clarify relationships between specialized use of wetlands and its probable impact on local diets.

## Regional context

**Fig 1:**
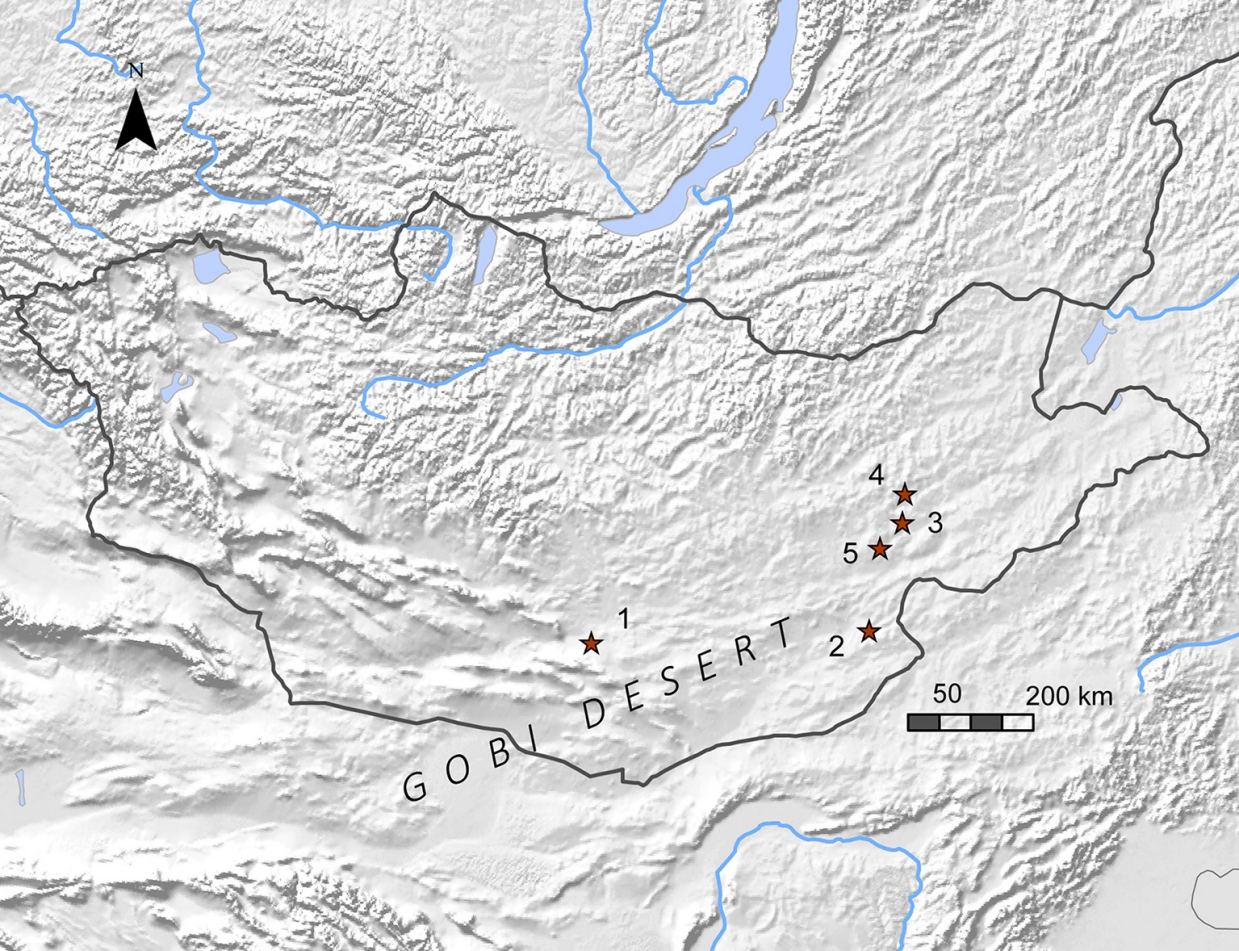
Map of Mongolia with sites mentioned in text: (1) Bayanzak/Shabarakh-usu, (2) Dulaani Gobi, (3) Zaraa Uul, (4) Delgerkhaan Uul, (5) Chandmani Khar Uul. Made with Natural Earth.

Very few stratified sites have been discovered, let alone excavated, in the Gobi Desert. The few previous excavations pre-date widespread use of chronometric dating in East Asia. The most notable of these is Bayanzak/Shabarakh-usu (Fig 1) in the southern Gobi Desert of Mongolia, discovered by Nels C. Nelson in 1925 and excavated by Mongolian and Soviet researchers in the mid-twentieth century [13–20]. Bayanzak/Shabarakh-usu is represented by extensive surface scatters, including several clusters associated with hearths. The environmental setting is characteristic of many Gobi Desert sites: extensive and sparsely vegetated dune-fields associated with a palaeolake basin, and within easy walking distance of both lowland steppe and upland plateau. Site assemblages are primarily Neolithic, spanning Oasis 2 and 3 (Table 1), and include artifacts from both the earlier and later periods. Dulaany Gobi (Fig 1), on the eastern edge of the Gobi Desert, was also discovered near an ancient lake basin and surrounded by mountains, clay terraces, and saxaul (*Haloxylon ammodendron*) [21]. Perlee and Ser-Odjav [21] discovered hearths surrounded by a microblade-based assemblage including projectile points, flakes, adzes and axes, grinding stones, a pickaxe, a spindle whorl, and ostrich eggshell, as well as copper or bronze slag. Retouched microblades were more common in lower levels, while bifacial flake tools and unifacially-flaked points were characteristic of the upper cultural level [22]. Researchers believed that the environment was characterized by wetlands, streams, and woodland at the time of occupation. Based on the assemblage they suggested that the site was occupied at different times during the fourth and second millennia BC and that inhabitants practiced a mixed economy of hunting and gathering, fishing, and low-level agriculture. More recent dating of sites with a similar range of tools suggests that the early component of this site would have dated to Oasis 2 (8000–5000 cal BP), while the later was probably associated with Oasis 3 (5000–3000 cal BP).

**Fig 2:**
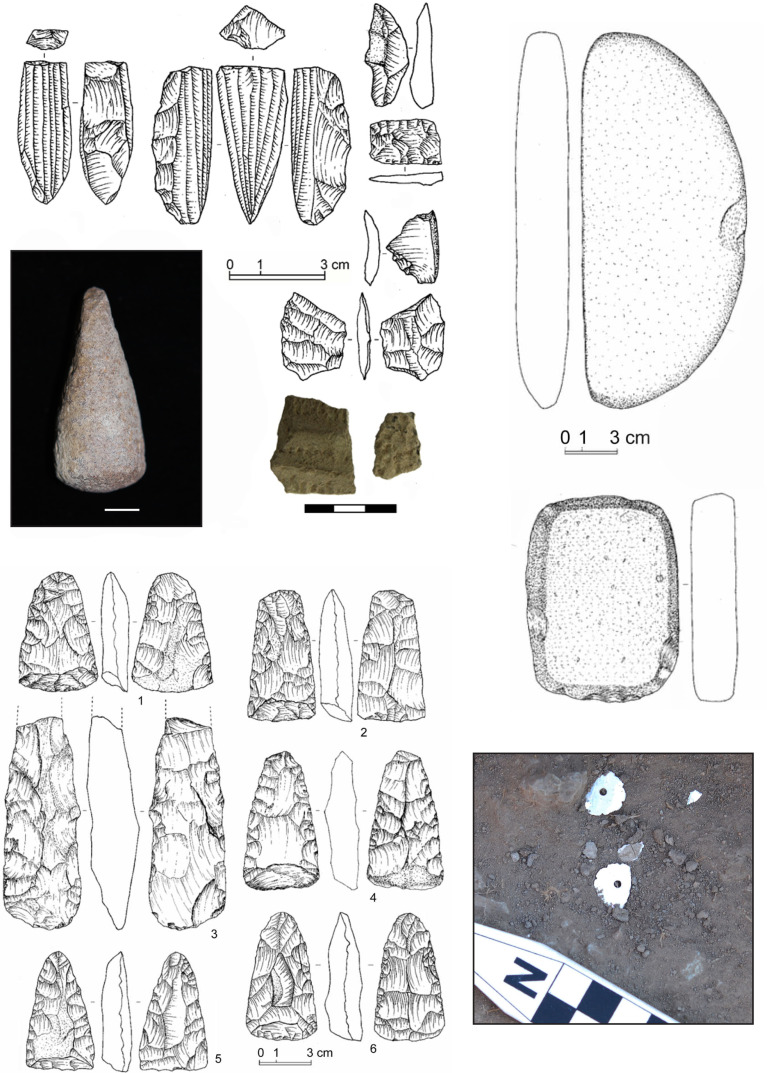
Examples of ground and flaked stone, pottery, and mother-of-pearl/nacre beads from Zaraa Uul.

The focus of our current study is Zaraa Uul (DMS 069 and 070), the first reported Gobi Desert site to be excavated since these early discoveries [22, 23]. Zaraa Uul was discovered by William Honeychurch and Joshua Wright in 2013 during survey of the area between Delgerkhaan Uul and Chandmani Khar Uul (Fig 1) and is located in the desert-steppe transitional zone of eastern Mongolia in the administrative district of Tuvshinshiree *sum*, Sükhbaatar *aimag*. The Zaraa Uul sites are concentrated between this basin and the eastern edge of a low north–south trending mountain range. The Zaraa Uul artifact assemblage is consistent with Oasis 2 (8000–5000 cal BP); it is dominated by microblade core reduction strategies, complimented by the use of flake cores, while unifacial and bifacial chipped projectile points, pottery, groundstone milling tools, and chipped adzes were all present (Fig 2).

**Fig 3:**
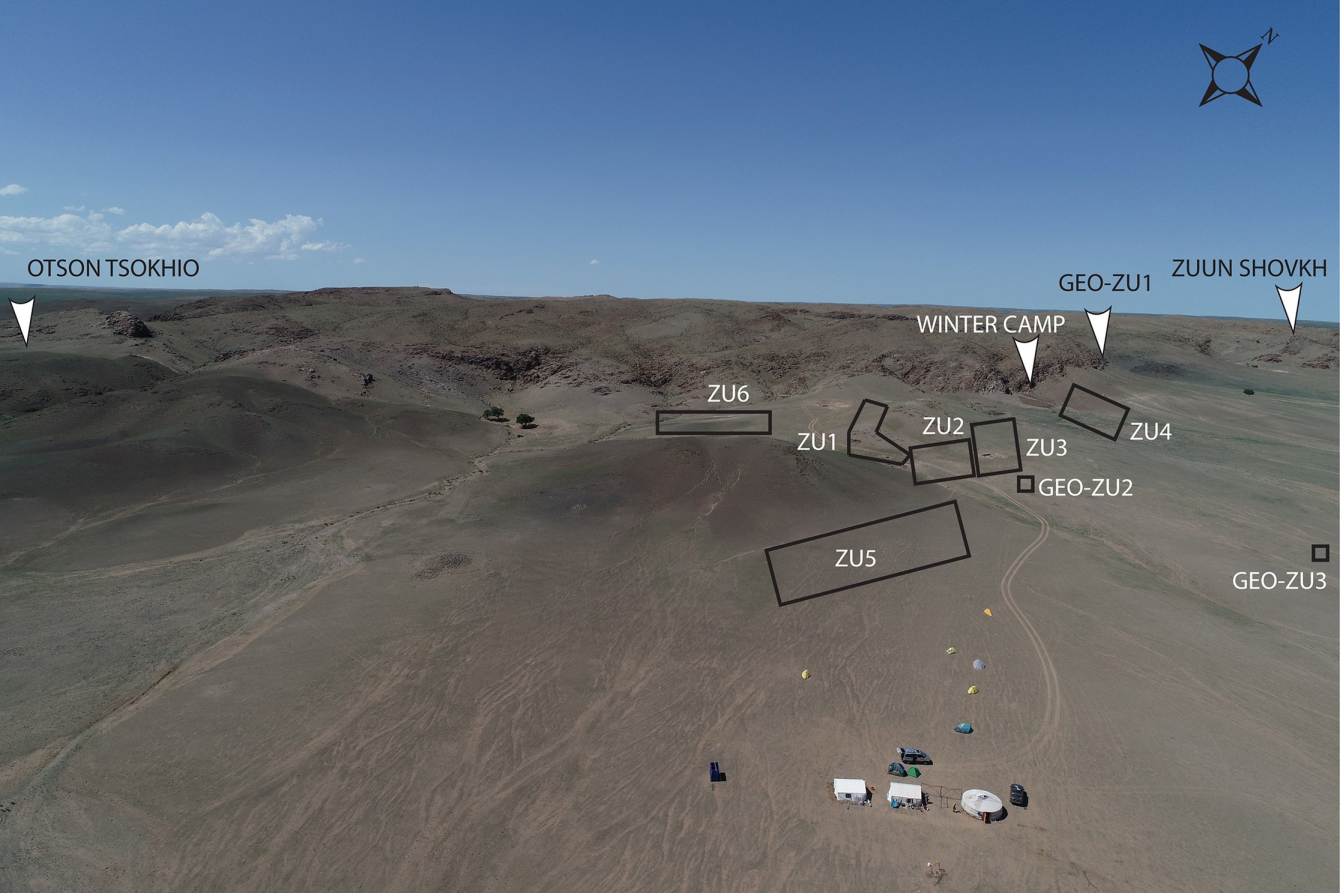
Aerial photograph of locality with location of sites and geopits. Drone image is property of Gobi-Steppe Neolithic Project, 2018.

Located about 220 km north of Dulaany Gobi, the natural environment is notably different from the dune-field/wetland oasis localities heavily occupied during this period. High density surface scatters are centred at the base of an eroded volcanic cone and adjacent felsite hills (Fig 3). The site sits at the edge of an extensive in-filled basin covered in herbaceous arid-adapted vegetation. The western margin of the basin is bounded by uplifted, folded terrestrial conglomerates and extrusive basalt flows while its floor is composed of incised Pleistocene marl from the remains of a vast saline paleolake that extended along its entire length [24]. Lithics consistent with the Early Upper Palaeolithic were discovered eroding from a Pleistocene beach ridge (Zuun Shovkh) approximately 2 km north of the volcanic cone in 2016 by D. Odsuren [25]. The sandy matrix of the excavation, consistent with beach sand deposits, were dated at the University of Washington Luminscence laboratory to 33,190–30,100 years (31.6 ± 1.59 ka) (Table 2). The discovery of artifacts within this beach deposit reveals a close association between human activity and a high stand of the lake during the OIS 3 Interglacial.

**Table 2:** Results of luminescence and radiocarbon dates for Zaraa Uul. Calibration was performed using OxCal v4.4.2 [31] and the IntCal20 calibration curve [46].

Laboratory ID#	Context	Artifact ID#	Material	Reported date	Calibrated years
					BP or ka
UOC-10163	Otson Tsokhio, Level 1	Ots.18.A1.L1:44	Bone collagen	29,514 ± 343	34,506–32,926 (95.4%)
UOC-10162	Otson Tsokhio, Level 2	Ots.18.A2.L2:29–32	Bone collagen	23,912 ± 161	28,363–27,697 (95.4%)
UOC-10164	Otson Tsokhio, Level 2	Ots.18.A1.L2:14	Bone collagen	25,609 ± 210	30,445–29,219 (95.4%)
UW3780	Zuun Shovkh	Zsh.16.GEO	sand	31,600 ± 1590	34,780–28,420 (95.4%)
UOC-9631	Locus 1, 70 cm below datum	T1-9a:114	Bone collagen	5358 ± 29	6276–6003 (95.4%)
UGAMS-22328	Locus 1, 66–77 cm below datum	T1-9g	Bulk ceramic	3230 ± 25	3495–3382 (89%)
UGAMS-22329	Locus 1, 76 cm below datum	T1-9j:207a*	Bone collagen	2580 ± 20	2753–2719 (95.4%)
UGAMS-22330	Locus 1, 80–86 cm below datum	T1-9m	Bone collagen	1850 ± 20	1863–1717 (95.4%)
UOC-7104	Locus 2, Level 1	T3.17.D.L1:13	Bone collagen	7607 ± 28	8441–8373 (95.4%)
UOC-7109	Locus 2, Level 1B	T2.16.B1.L2:1	Bone collagen	1523 ± 20	1520–1350 (95.4%)
UOC-7107	Locus 2, Level 1B	T3.17.E1.L1B:x	Bone collagen	6883 ± 29	7788–7665 (95.4%)
UOC-7105	Locus 2, Level 1B	T3.17.B.L1:13	Bone collagen	6619 ± 28	7570–7457 (93%)
UOC-7106	Locus 2, Level 1B	T3.17.E.L1:10	Bone collagen	7344 ± 33	8208–8030 (91.0%)
UOC-7100	Locus 2, Level 2	T2.16.D1.L2:7	Bone collagen (*H*. *Sapiens*)	7089 ± 30	7971–7850 (95.4%)
UOC-7108	Locus 2, Level 2	T3.17.B.L2:13	Bone collagen	7342 ± 37	8212–8026 (89.8%)
UOC-7110	Locus 2, Level 2	T3.17.F.L2	Bone collagen	7117 ± 31	8055–7924 (80.4%)
UOC-7112	Locus 2, Level 2	T2.16.D2.L4:2	Bone collagen	7276 ± 37	8171–8014 (95.4%)
UGAMS-22331	Locus 2, Level 2	T2-3b:38	Bone collagen	6990 ± 30	7878–7736 (78.2%)
UGAMS-22332	Locus 2, Level 2	T2-3e:65a	Bone collagen	6620 ± 30	7571–7442 (95.4%)
UGAMS-22333	Locus 2, Level 2	T2-3g:1	Bulk ceramic	6580 ± 30	7515–7430 (85.1%)
UGAMS-22334	Locus 2, Level 3	T2-3m:1a	Bulk ceramic	5910 ± 30	6794–6665 (95.4%)
UOC-7103	Locus 3, Level 1	T1.17.B2.L1:3	Bone collagen	2412 ± 22	2491–2353 (95.4%)
UOC-7101	Locus 3, Level 2	T1.17.A1.L1B:23	Bone collagen (*P*. *tigris*)	5765 ± 25	6639–6493 (95.4%)
UOC-7102	Locus 3, Level 2	T1.17.C2.L1B:24	Bone collagen	6111 ± 27	7156–6894 (95.4%)
UW3939	Locus 3, Level 2	T1.17.GEO	Sediment (IRSL)	4640 ± 810	5450–3830 (95.4%)
UOC-4480	Geological	GEO-ZU3_2b	Bulk sediment	5534 ± 31	6399–6288 (95.4%)
UOC-4481	Geological	GEO-ZU3_2b	Bulk sediment (humic)	3651 ± 29	4013–3890 (70.8%)
UOC-4482	Geological	GEO-ZU3_2b	Bulk sediment (humin)	5547 ± 27	6399–6296 (95.4%)
UW3684	Geological	GEO-ZU3_2a	Sediment (IRSL)	6700 ± 300	7300–6100 (95.4%)
UW3685	Geological	GEO-ZU1	Sediment (IRSL)	6900 ± 300	7500–6300 (95.4%)

**Fig 4:**
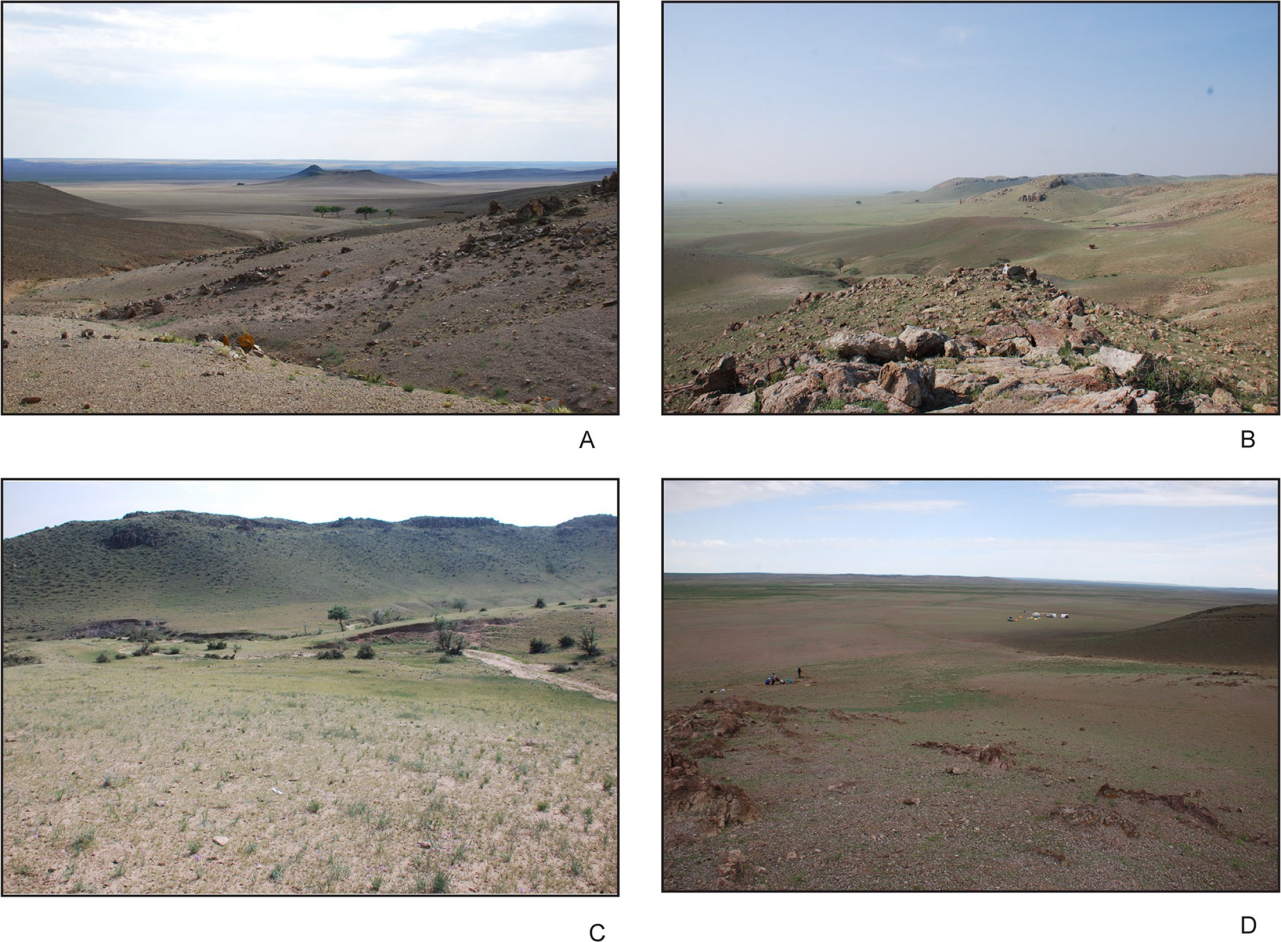
Landform morphology across different sections of the basin: (A) view of basin from Otson Tsokhio, (B) view of basin and ranges looking towards Zaraa Uul from Zuun Shovkh, (C) view of landscape around stream sampled for GEO-ZU1, and (D) overlooking Zaraa Uul loci from the saddle of hill with the extinct volcano in the upper right of image.

The remnant marl of the former lake currently forms hummock and terrace lines where it was incised by streams flowing down from alluvial fans on the margins, and bisected in length by a larger ephemeral stream (Fig 4). In many places these hummocks are covered with coarse-grained alluvium washing out from the surrounding hills after storms [24]. Today these drainages are dry for most of the year. There is at least one active freshwater spring at the south end of the basin, forming a pool and stream where livestock drink and bathe. At the east side of the basin, about 1.5 km south of Zaraa Uul, there is a small salt marsh with saltwater ponding. The excavations at Zaraa Uul have already produced information that holds substantial promise for understanding palaeoenvironmental change in arid East Asia and contributing to our understanding of Neolithic chronology. This report relates our archaeological findings to previous knowledge of Gobi Desert chronologies and builds an initial framework for the chronology of palaeoecological change in the far eastern steppe.

## Materials and methods

### Excavation and survey

Excavation was conducted under permits from the Ministry of Education, Culture, Sports and Science through the Institute of Archaeology, Mongolian Academy of Science (#15-1-19, #16-1-06, #17-1-05, #18-1-3/3). Work included small-scale excavation of two Upper Palaeolithic habitation sites (Otson Tsokhio and Zuun Shovkh) and we excavated six Bronze Age burials, but the primary focus was Neolithic habitation. These Neolithic habitation sites are in close proximity to Bronze Age burial structures first used during the Ulaanzuukh period [26–28]. Many burial structures were recorded stretching across the eastern face of the mountain range, but most evidence for habitation relates to Oasis 2 of the Neolithic period [22]. Habitation appears to have been heavily localized. Within the main area of occupation there are several overlapping scatters of lithic assemblages spanning the lower edge of the hillslope, along the saddle of the hill, and behind (west) of the eroded volcanic cone (Fig 3). The nearby presence of a modern pastoralist winter camp (Fig 3) suggests that this area provides good protection from north winds during the winter. Conversely, the site is heavily affected by wind during the summer, which may have provided desirable protection from mosquitoes and other insects during wetter climatic phases.

**Fig 5:**
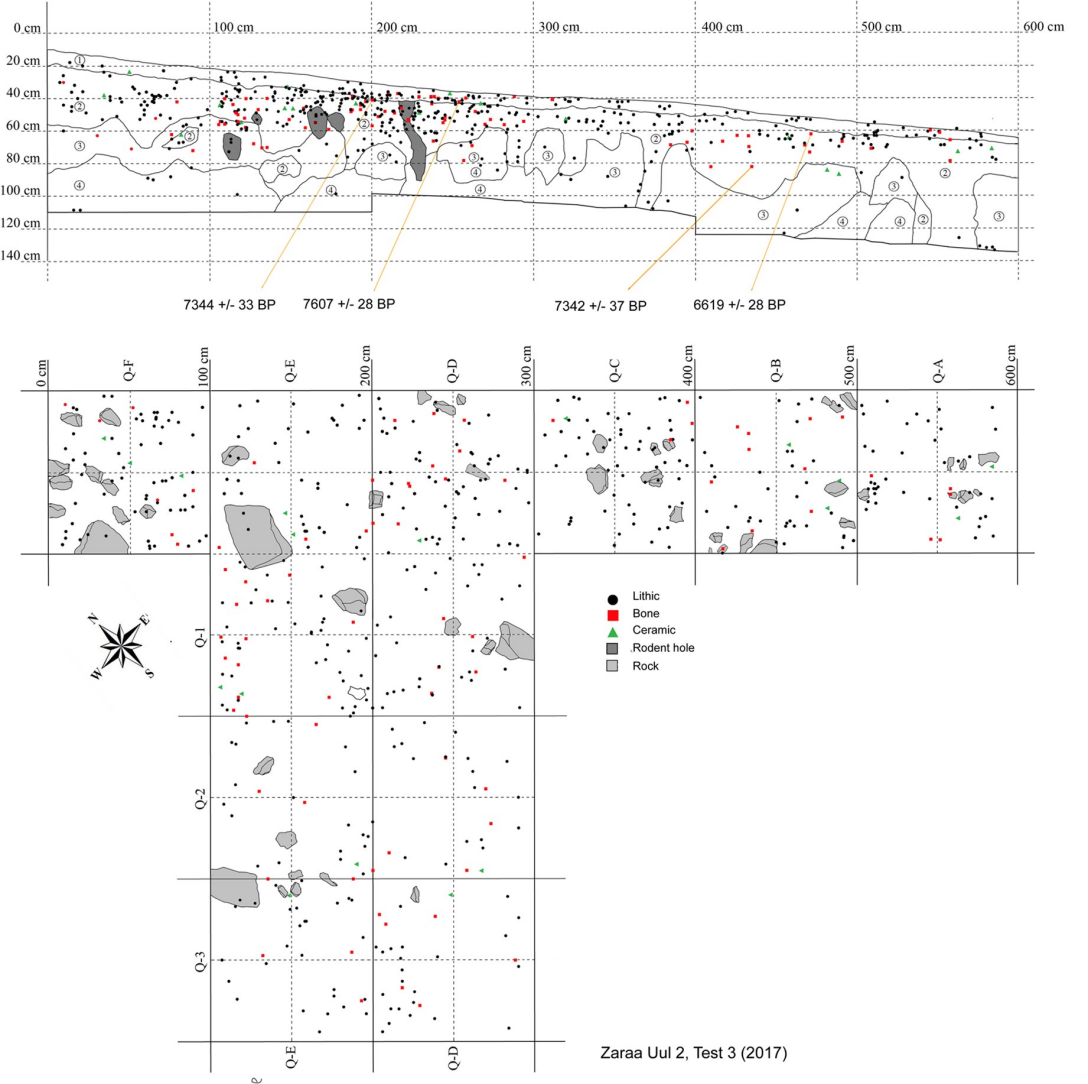
Map of 2017 excavation at Zaraa Uul, Locus 2 (ZU2.T3).

**Fig 6:**
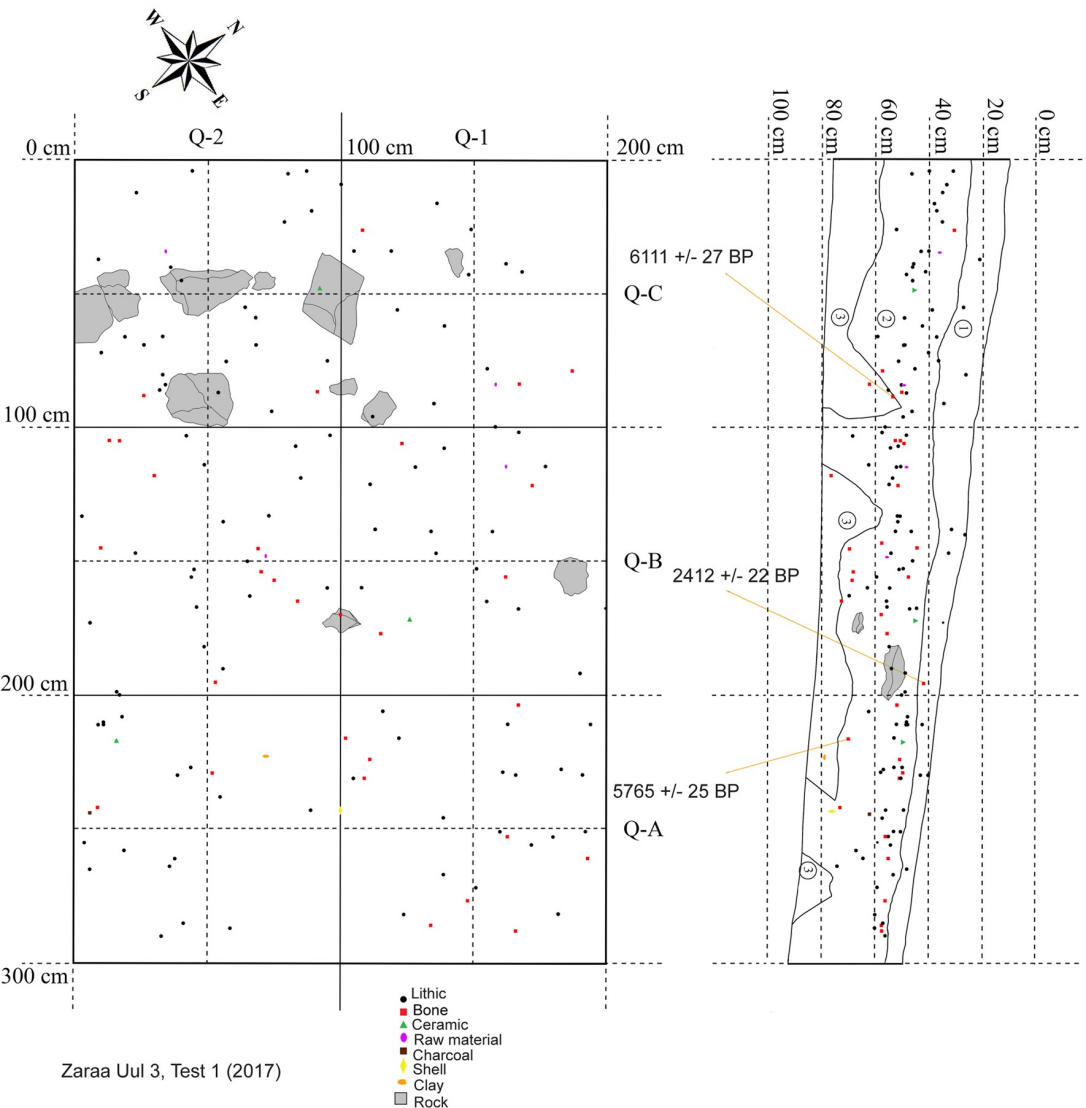
Map of 2017 excavation at Zaraa Uul, Locus 3 (ZU3.T1).

Our preliminary mapping of the primary lithic scatters divided Zaraa Uul into four subsites (Fig 3). Excavations focused on Zaraa Uul 2 (ZU2) and Zaraa Uul 3 (ZU3), where surface artifacts were most densely concentrated. Zaraa Uul 2 represents an area of the surface with especially high artifact density and is located on the eastern abutment of the volcanic cone. Subsurface artifact density was determined by augur test pits and 0.5 m x 0.5 m shovel test pits at multiple elevations across the slope and saddle of the hill. Variation in both surface and subsurface artifact density was used to determine placement of test units. Locus 1 (ZU2.T1) was chosen based on the relative absence of surface and presence of sub-surface remains, with the hope that this would translate into better preservation. Locus 2 (ZU2.T2) was chosen based on very high artifact density in both the surface and sub-surface components, in addition to well-preserved faunal remains. We excavated a 1 m x 2 m unit in Locus 1 and a total of 26 m^2^ in Locus 2 (ZU2.T2, ZU2.T3 or ZU.T2/3) (Fig 5). Locus 3 (ZU3.T1) is represented by Test Unit 1 within ZU3 (Fig 6). Artifact scatters were visibly less dense on the surface. Placement of Locus 3 was determined based on 0.5 m x 0.5 m shovel tests positioned every 6 m moving northeast along the base of the hill. We placed ZU3.T1 (“Locus 3”) directly above a test unit with a denser concentration of bone and stone artifacts (ST.17.F6), which contained a canid (*Vulpes* sp.) tooth, a large cylindrical microblade core and broad microblades. This scatter had fewer surface artifacts than ZU2.T2/3 (“Locus 2”), overlaps with the eastern edge of Zaraa Uul 2 and is located directly downslope from the saddle of the main hill (Fig 3).

**Fig 7:**
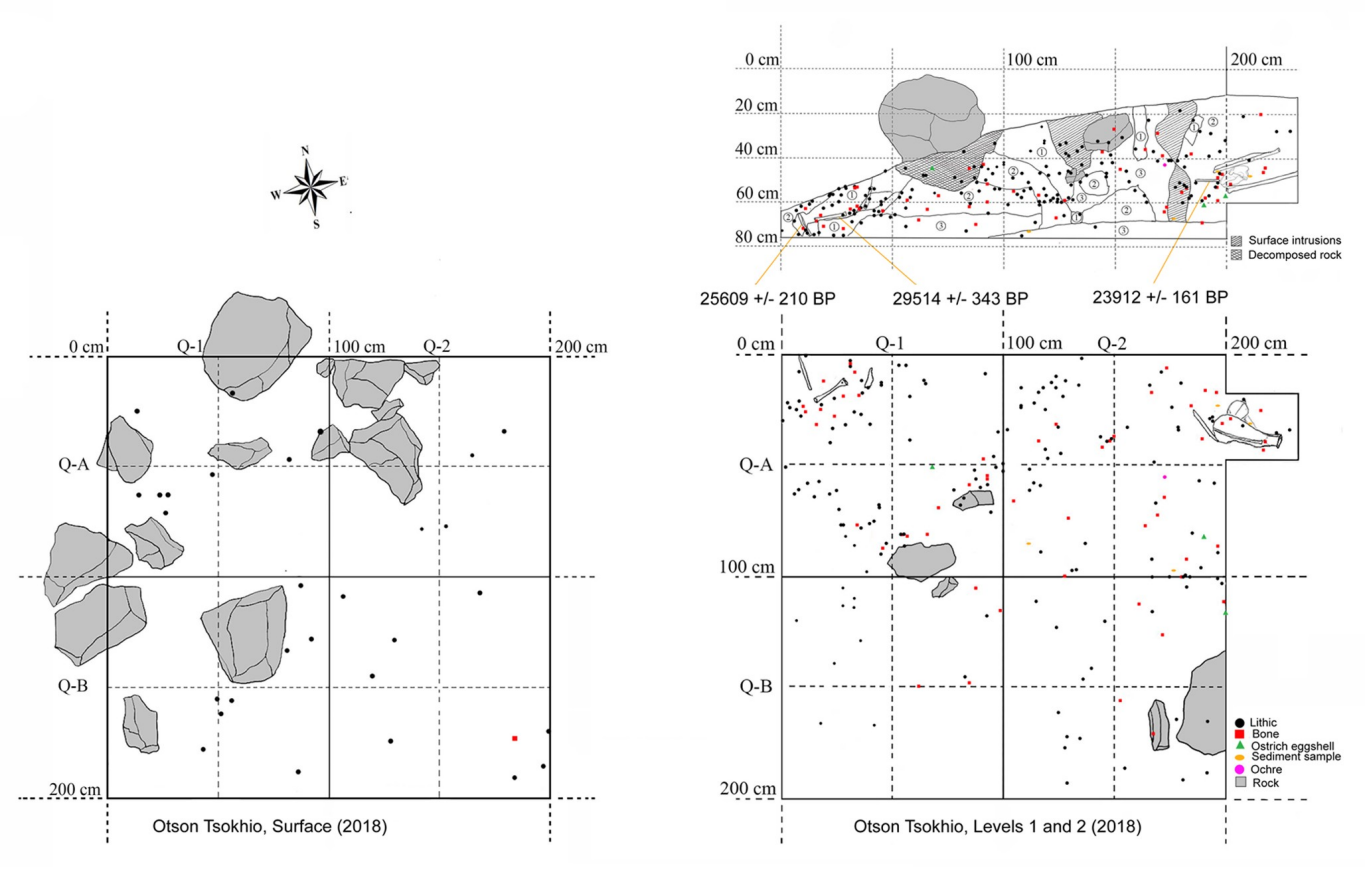
Map of 2018 excavation at Otson Tsokhio.

At Otson Tsokhio, a 2 m x 2m unit was laid out directly over an area where the densest deposit of equid bones and stone tools were seen weathering from surface matrix, primarily around several partially buried boulders (Fig 7). We uncovered a deposit of well-preserved bone (*Megaloceros* sp. [giant elk] metatarsal, *Coelondonta antiquitatus* [woolly rhinoceros] scapula and juvenile Hyaenidae [hyaena] skull) on the north-east wall and extended the unit to recover these (Fig 7). Ostrich eggshell fragments and one ostrich eggshell bead were also recovered. A 2 m x 2 m test unit was excavated at Zuun Shovkh in what appeared to be a relatively undisturbed area. The matrix was primarily sand and few bones or cultural remains were recovered. All of the few bones recovered were bagged together.

All sediment from all these excavations was sieved using 3 mm mesh. Catalogue numbers were assigned as follows: subsurface scatter number, year of excavation, test unit number, grid number, level and identifier (i.e., “ZU3.18.T1.A2.L2:1”). Each grid was laid out on a north-east to south-west axis, set parallel to the orientation of the local basin-range landforms; the x-axis was labelled at 1 m intervals with numbers and the y-axis was labelled at 1 m intervals with letters. Small bone and seemingly undiagnostic fragments were collected, along with bone from the sieve, in a common grid bag with the catalogue number and the name “bone” rather than an individual number (i.e., “ZU3.18.T1.A2.L2:bone”). Artifacts and diagnostic or larger bone fragments were mapped in three dimensions within the grid using a string, line level and datum (10 cm above ground surface). In order to track changes in elevation within the excavation, numerous elevation points were collected within the grid using the datum. Primary levels (e.g., Level 1, Level 2) were determined based on matrix characteristics (i.e., Munsell colour, granular structure, and soil textural class), beginning with Level 1 uppermost. Within deeper matrices, sublevels were set at approximately 10 cm intervals within the main level (e.g., Level 1a, Level 1b), with the exception of 2018 excavations at Locus 3 where sublevels were placed at 5 cm intervals in Level 1. The above procedure was followed for all units.

### Landscape geomorphology

Geoarchaeological research at this site was led by Rosen and included walking observations, excavation and sediment collection from three geological test pits (Fig 3) around the margin of the basin. Observations on a number of high beach and stream terraces surrounding the former lake basin were used to understand the Quaternary geomorphological history of the locality. Detailed methods and results are being prepared for a forthcoming publication [24].

### Chronometric dating

Samples for AMS ^14^C were taken from multiple levels of loci 1–3. Pottery and bone collagen were processed at the University Georgia Center for Applied Isotope Studies and the A. E. Lalonde AMS Laboratory. Collagen for all samples sent to the A. E. Lalonde AMS Laboratory was processed using ultra filtration by Janz at the Trent Environmental Archaeology Laboratory (TEAL) according to the UCI-KCCAMS procedure for ultrafiltration [29]. Bulk ceramic samples were processed based on the guidelines outlined in [30]. One bulk sediment sample was processed at the A. E. Lalonde AMS Laboratory following standard laboratory protocol [31]. The A. E. Lalonde AMS Laboratory analyzed the bulk fraction and humin fraction of sediments along with humic acid extraction as a cross-check. Calibration was performed using OxCal v4.4.2 [32]. Luminescence dating was carried out at the University of Washington Luminescence Laboratory.

### Phytoliths

During the archaeological season of 2015 at Zaraa Uul, the excavation team collected 24 sediment samples from loci 1 and 2 for both sedimentological and phytolith analyses. The samples included 19 which came from archaeological test pit excavation sample points, and five control samples from off-site contexts within the natural alluvial deposits of a nearby seasonal-stream. These samples were analysed by Dr. A. M. Rosen and her research team at the Environmental Archaeology Laboratory, Department of Anthropology, University of Texas at Austin. Phytoliths were extracted according to the laboratory’s standard protocol using decalcification, gravity sedimentation to remove clay particles, dry-ashing to remove organic matter, and heavy liquid flotation. A detailed description of the procedures for extraction and quantification has been published in [33].

### Fauna

Analysis of archaeological fauna from loci 1 and 2 was carried out by Janz using the comparative collection in Eugène Morin’s Archaeozoology Laboratory, Department of Anthropology, Trent University. Faunal analysis served two primary purposes: a) as direct evidence of human diet; and b) to better understand the palaeoecology during site occupation. Janz developed a list of possible prey species based on published data on Mongolian mammalian [34] and avian [35] fauna. The lack of access to a comparative collection for Far Eastern desert and steppe small mammalian and avian faunas proved challenging for several small-bodied species; however, the range of genera present among local wild fauna is consistent with Eurasian Pleistocene and North American steppe species. Avian fauna were rare, but could often be identified to Order or Family.

All mammalian fauna were divided into size classes [S1 Table], based on published body weight ranges of the species included in this analysis: mega mammal or “MGMAM” (> 1000 kg); large mammal or “LMAM” (> 150 kg); large medium mammal or “LMMAM” (50–150 kg); medium mammal or “MMAM” (10–50 kg); small mammal or “SMAM” (2–10 kg); and micromammal or “MCMAM” (< 2kg). Large medium mammals were classified as large mammals to reduce categorization errors during quantitative analysis. When size was uncertain between two class (“SMMAM”), the larger size class was selected (“MMAM”). As such, the quantitative data presents a systematic bias towards larger size classes.

Unidentifiable ungulates were classified according to size: “LUNG” (> 150 kg), “MUNG” (50–150 kg), and “SUNG” (10–50 kg). Cervids and bovids were seldom identifiable beyond Order and were categorized according to the same size classes used for all ungulates. There is some overlap in size categories with ungulate species, particularly between medium and small size classes. For example, small female caprines would fit in the upper limits of the small bovids or medium mammal categories while large males would be considered medium bovids or large medium mammals.

Categorizations of both “large bovid” and “Bovinae” (*Bos* sp. or *Bison* sp.) were made during laboratory analysis, but these were grouped under Bovinae during quantitative analysis. All Bovinae elements were checked against comprehensive published studies of the post-cranial morphological differences for European [36] and American [37] species. Elements clearly recognizable as belonging to *Bos* sp. in this study included ulna, tibia, metapodials, carpals, phalanges. None were identified as *Bison* sp. All Neolithic-period habitation (8500–6500 cal BP) at Zaraa Uul predates the arrival in East Asia of *Bos taurus* and other domesticated herd animals by more than a millennium [38]. As such, all Bovines in this study likely belong to *Bos primigenius*, though the possibility of *Bison* spp. are not excluded.

Carnivore sizes were classed as follows: “LCARN” (> 25 kg), “MCARN” (2–25 kg), and “SCARN” (< 2 kg).

There is always some chance that faunal assemblages include intrusive elements, particularly in situations were there is the level of rodent activity and evidence of post-depositional bioturbation outlined for the Holocene layers of Zaraa Uul (see “Geomorphological Context”). The calcium-rich context of these burials has caused extensive mineralization on the surfaces of most artifacts and bones. This is especially true of the Holocene layers. There are no studies relating to the timeline for surface mineralization in these sites, especially as it might relate differently to large ungulate versus less porous bone such as that of small avian fauna. Comparison between level of surface mineralization on Early-Middle Holocene fauna and actual dates was inclusive: dates did not match expectations based on relative degree of mineralization. However, bones with no evidence of surface mineralization visible to the naked eye were categorized as intrusive (N = 6) and although they are reported in Table 5, they were excluded from quantitative analysis. Those with moderate levels, comparable to poorly mineralized Early-Middle Holocene-dated fauna, were included (N = 4 [62 specimens from *Marmota sibirica* are counted together]). All were derived from Locus 2 (ZU2.T2/3) with the exception of the one likely intrusive from Locus 3 (Rodentia femur), and one possible intrusive from each Zuun Shovkh (Cricetidae tibia) and Otson Tsokhio (Rodentia tooth) (see S2 Table).

## Results

### Geomorphological context

The highest of the observed beach terraces was recorded at an approximate elevation of 1044 m a.s.l. (Garmin GPS). A large lithic scatter eroding from the whitish/yellow silty sand deposits of this ridge, Zuun Shovkh, are typologically consistent with the Initial to Early Upper Palaeolithic in Mongolia (ca. 45,000–26,000 cal BP [39, 40]. The luminescence date on the sand matrix, dated at 31,600 ± 1590 years (UW3780) (Table 2), is consistent with wet-phased glacial expansions in the Altai Mountains in western Mongolia [41]. Radiocarbon dates on collagen from the nearby Otson Tsokhio date at ~34,100–27,700 cal BP (Table 2). We thereby date the two sites to a prolonged period of high lake stands across arid East Asia during at ~50,000–23,000 cal BP during the MIS 3 interglacial [41–45]. At large saline lake would have filled the entire basin. A subsequent drop in the lake level and ensuing erosion of the upland streams as they adjusted to the lower base level, led to the deposition of yellowish sand beaches along the foot slopes of the bedrock hills ringing the basin, as observed at the base of GEO.ZU2 [24]. These deposits represent the last high stand of the Pleistocene saline lake, which may have existed until as late as the warmer and moister Bølling/Allerød (ca. 15,400–13,000 cal BP [after 44]). The side valleys draining into the lake basin filled with alluvium and beach sand throughout this period during minor transgressions and regressions of the lake. The upper levels of the terminal Pleistocene beach deposit contain weak soil development, appearing as a darker brown, siltier unit capping the Late Pleistocene beach sands and contemporary alluvial facies from nearby stream valleys (e.g. GEO.ZU1) [24]. This period of accumulation was followed by a severe drop in lake level, probably coinciding with the Younger Dryas arid phase (ca. 13,000–11,500 BP [after 44, 45]) that may have dried up most of the lake basin. Former Pleistocene beach deposits and the exposed marl of the former lake basin were deeply incised, leaving hummocks of marl and deeper swales between them [24].

**Fig 8:**
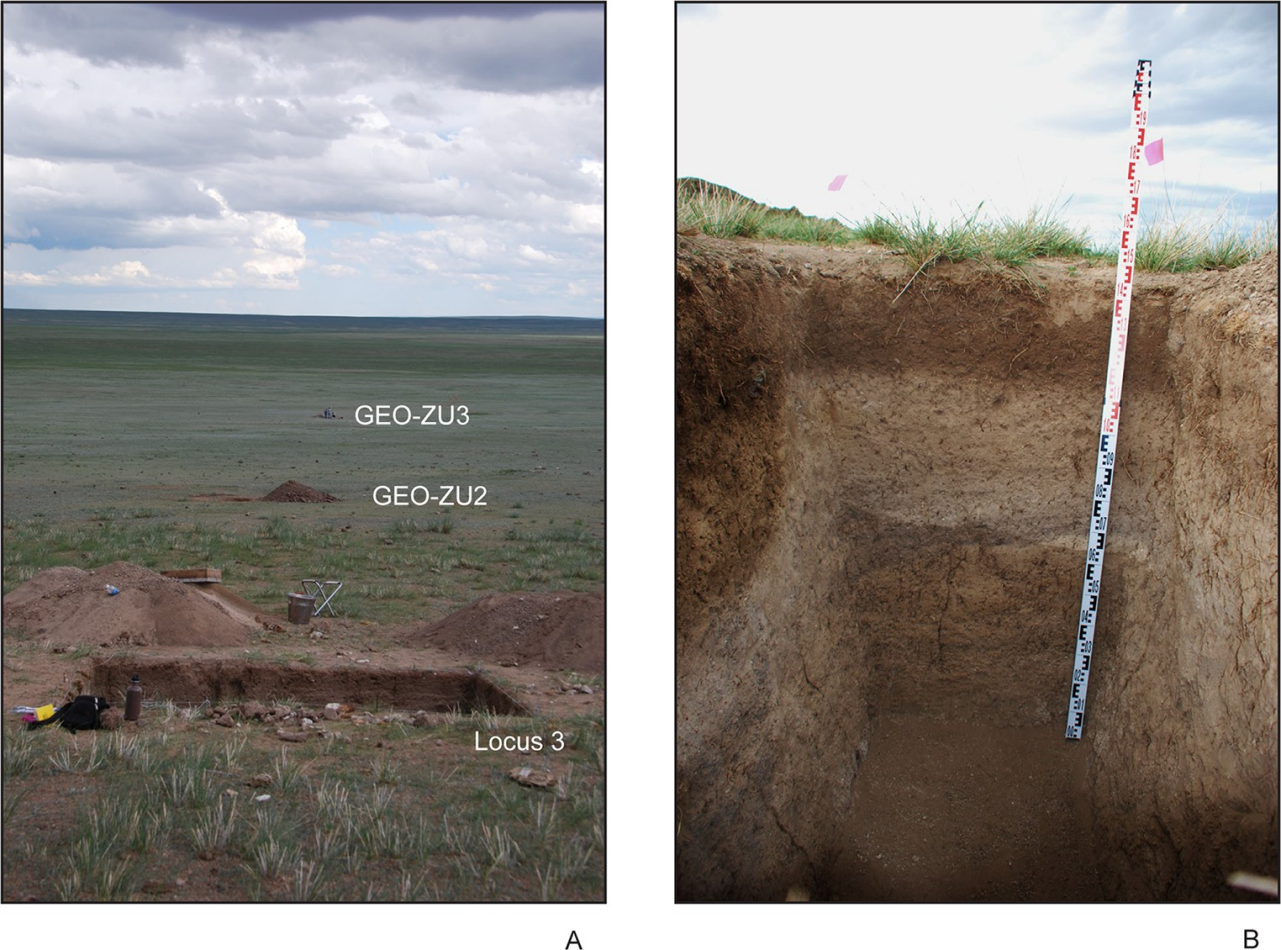
(A) Excavation of Locus 3 (ZU3.T1) and location of geopits GEO.ZU2 and GEO.ZU3 relative to Locus 3. (B) view of intact stratigraphy at Zaraa Uul.

Most relevant to this study is the return to warm/humid conditions, particularly during the Middle Holocene Climatic Optimum (ca. 8000–4000 cal BP), during which time the East Asian Summer Monsoon System (EASM) brought heightened precipitation far into Northeast Asia [47–51]. At Zaraa Uul this period manifested itself with the resumption of strong alluvial activity under a regime of increased rainfall events. This period of geomorphological activity is evident in GEO.ZU1, with evidence of renewed stream activity evidenced by well-sorted stream-channel pebbles, floodplain build-up, and floodplain back-swamp formation from overbank flooding [24]. Luminescence dates from GEO.ZU1 indicate that these floodplains were stable and well-developed at 6880 ± 260 years (Table 2). On the basin plain, increased rainfall and outwash filled the swales that had been sculpted into the marl from previous erosional episodes. These swales were transformed into fresh-water seasonal marshes. This process is evidenced in GEO.ZU3, southwest of our archaeological test units and located farther into the former basin (Fig 8A), where we recorded a one-meter build-up of heavy clays with very high concentrations of dark organic matter. The black clay unit in this section contains large well-developed CaCO_3_ nodules, indicating that these ponds were seasonally dry [24]. One luminescence and three bulk sediment radiocarbon dates were taken on sediment sample (GEO.ZU3.2a and 2b) from this deposit. The sediments were dated by luminescence to 6690 ± 270 years (Table 2). The acid wash treatment of bulk sediment produced a date of 6398–6288 cal BP (95.4%), while the humin fraction produced a date of 6398–6295 cal BP (95.4%) (see Table 2). The humic fraction was dated to 4013–3889 cal BP (71.1%) suggesting that these ponds were active until the end of the Middle Holocene humid phase, and then transformed to patches of moist humic soils before drying completely. The Late Holocene was marked by a return to arid conditions after ~4300–4000 BP and continuing into the present [33, 48, 52].

Level 1 of Zaraa Uul Locus 2 (ZU2.T2/3) and Locus 3 (ZU3.T1) extends ~5–10 cm below the surface and is characterized by recently re-deposited sediments–a poorly consolidated mix of aeolian deposits and slope wash. Redeposition of artifacts from slightly farther upslope in Locus 2, Level 1 would explain both the lack of spatial patterning (Fig 5) and the fact that the earliest date is from Level 1 (8442–8374 cal BP [7607 ± 28]; see Table 2). Burrowing rodents were likely also responsible for movement of artifacts through the matrix; there is clear evidence of disturbance in areas of Locus 2 (Fig 5) and in Locus 3, Quadrant A-2 (Fig 6), where a nearly complete post-cranial skeleton of a hare with small carnivore toothmarks was recovered from. We interpreted this as a predator (fox) cache and this individual was excluded from quantitative analysis.

Level 2 extends ~11–50 cm below surface and is characterized by a darker A-horizon which represents several thousand years of Holocene soil development under stable landscape conditions. A potsherd (T2-3m:1a) from Locus 2 at the Level 2/3 boundary was dated to 6794–6665 cal BP (5910 ± 30) (Table 2), but recovered from an area disturbed by rodent burrowing. The luminescence date from Level 2, Locus 3 (34 cm below surface) provided direct dates of 5450–3830 years (4.64 ± 0.81 ka on younger component [63%], 6.01 ± 0.47 for central age model) from near the middle of that level (Table 2).

Level 3, ~25–50 cm below the surface, represents the B-horizon. It contains few artifacts and is characterized by hard grayish deposits with high levels of CaCO_3_ derived from the older Pleistocene marls. Levels 2 and 3 were heavily disturbed and intermixed in Locus 2, but can be characterized by distinct changes in colour and texture. Level 3 was less well-developed in Locus 3. Artifacts in Locus 3 were primarily distributed in Level 2 at 30–50 cm below the surface (Fig 6), placing primary occupation in this section of Zaraa Uul at about 6500–7100 cal BP during a period of *in situ* soil development and stable landscape conditions (Table 2). Level 3 was underlain in both contexts by gleyed sediments indicating ponding, which in turn overlay the yellow beach sands, and more gleyed sediments below. These layers record successive wet and dry phases in which the lake transgressed and regressed. Non-diagnostic flint artifacts and nodules were recovered discontinuously throughout these layers.

### Chronology of occupation

We identified two primary periods of intensive habitation around Zaraa Uul. In addition to the Middle Holocene component comprising Zaraa Uul, we discovered two Early Upper Palaeolithic occupations, all within a 3 km radius. One luminescence date on the Zuun Shovkh sand deposit, and radiocarbon dates from Otson Tsokhio indicate that these localities, all within 2 km of Zaraa Uul, and 3 km of one another, were occupied during the Early Upper Palaeolithic. This phase of habitation, at ~34,100–27,700 cal BP corresponds with heightened humidity and high lake stands across arid East Asia during MIS 3. The most intensive phase of Holocene use likewise corresponds to a period of high humidity. Radiocarbon and luminescence dates on darker clayey sediments indicate that the locality experienced a wet phase during the Middle Holocene which lasted until at least 6690 cal BP and possibly tapered off until ~4014–3891 cal BP.

Auguring, shovel tests, and excavation all indicate that Holocene occupation of the area around Zaraa Uul commenced at ~8500 cal BP with the onset of climatic amelioration. Human activity was initially focused on hard Pleistocene sediments (Level 3) which provided a hard, sparsely vegetated surface for camping and working. Site activity after 8500 cal BP contributed active anthropogenic disturbance of these sediments during the formation of Holocene soils and was responsible for the intermixing of levels 2 and 3 at Locus 2 (ZU2.T2/3). Site occupation over the next two millennia resulted in the mixing of occupation debris, as did rodent activity and processes of erosion and redeposition during the more arid conditions of the Late Holocene. Radiocarbon dates on bone and ceramics consistently, with only two exceptions, predate 6500 cal BP. One date from Locus 1 shows continued human activity at 6276–6003 cal BP. Regular use of the site appears to end *during* the period of *in situ* soil development, but around the same time as the onset of drying in freshwater marshes beginning by ~6400 cal BP.

Following what appears to be a hiatus of about three thousand years, the locality was used in Bronze Age mortuary rituals. There are several later periods of discontinuous activity represented: 3555–3382 cal BP (3230 ± 25) in Locus 1, 2753–2353 cal BP (2580 ± 20, 2412 ± 22) in loci 1 and 3, 1520–1350 cal BP (1523 + 20) in Locus 2, and 1863–1717 cal BP (1850 ± 20) in Locus 1. The first two phases correspond with periods of known Bronze Age burial activity (Ulaanzuukh and Slab Grave periods [26]) upslope from the site. The presence of burials and less intensive evidence of habitation than in the earlier period, suggests that the valley was occupied after ~3500 cal BP but that this particular location on the landscape no longer suited the needs of local people.

### Phytoliths

Phytoliths were analyzed at Zaraa Uul to investigate a) the diversity of micro- environments around the site which were available to be exploited for different types of resources on the part of the Early and Middle Holocene populations who visited this locality, b) to address questions of seasonality of occupation, and c) to help contextualize the environmental background for the faunal remains at the site. All samples came from the Early-Middle Holocene contexts in loci 1 and 2 (see below).

**Fig 9:**
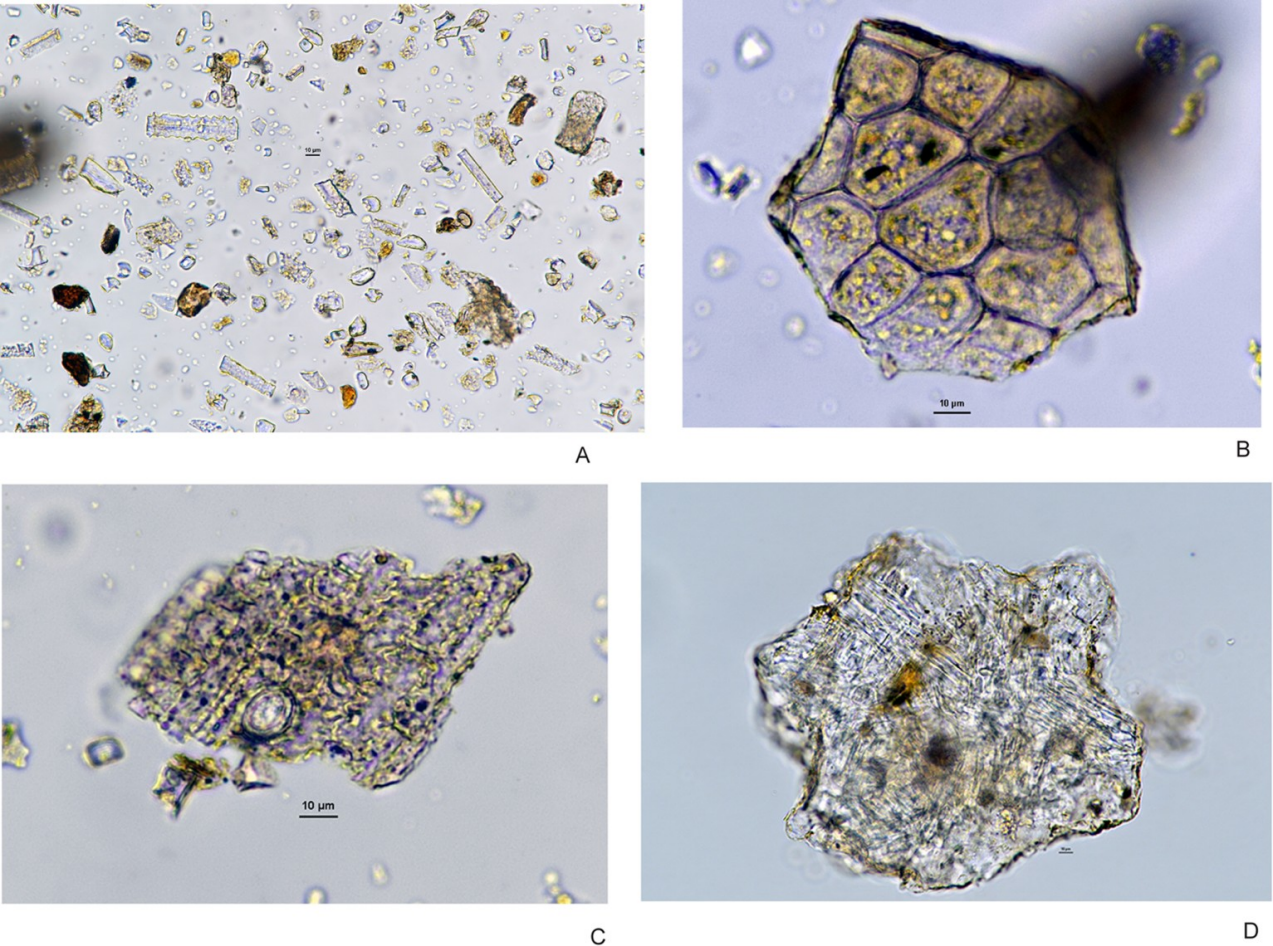
Examples of Zaraa Uul phytoliths: (A) Poaceae shoots and leaves, (B) dicot leaf, (C) Cyperaceae stem, (D) succulent.

**Fig 10:**
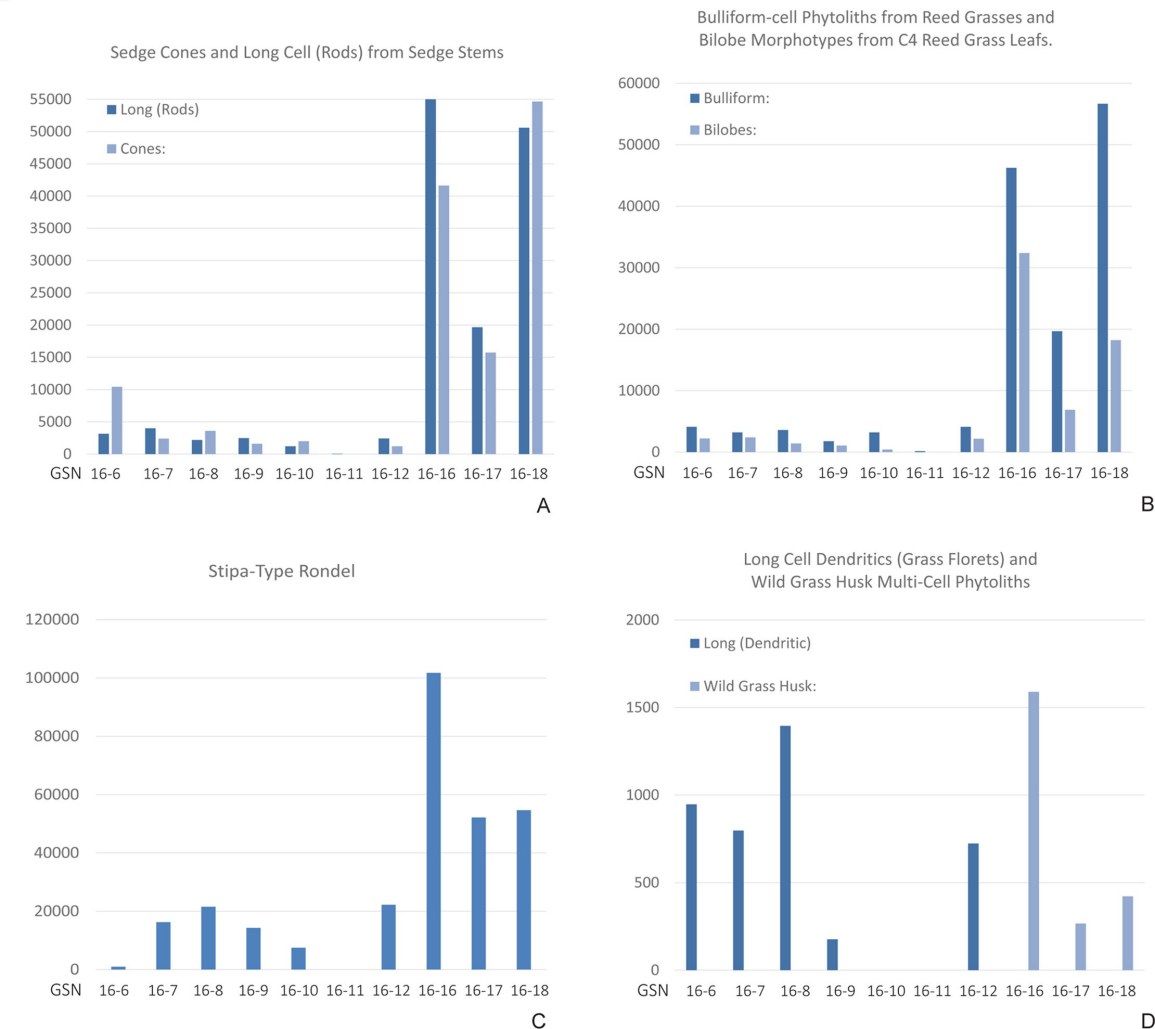
Visual comparison between numbers of sedge, reed, and grass phytoliths from each sample. Locus 1: 16–16, 16–17, 16–18. Locus 2: 16–6, 16–7, 16–8, 16–9, 16–10, 16–11, 16–12.

Here we report on the counts of phytolith samples from ten samples. The results are listed in Table 3. They are summarized as follows: the samples are dominated by phytoliths from genera of grasses (Poaceae), including reed grasses, with some morphotypes from sedges (Cyperaceae) (Fig 9C). For the most part, the phytoliths of the grasses and sedges come from the stems rather than the floral parts of the plants (Fig 9A), although there are some floral parts are present in both sets of samples (Fig 10D). Depending on the species, local grasses flower from late June into August and seed through late July into September [53], suggesting that primary occupation occurred in summer/fall months while the grasses were flowering and seeding, or inhabitants were using stored seeds over winter months. The presence of waterfowl and hibernating species such as marmot (*Marmota sibirica*) and badger (*Meles leucurus*) in Locus 2 (see below) support a summer or fall occupation. Some of the phytolith forms are from the leaves and wood (single cell, platey) of woody shrubs or trees (Fig 9B). These plants (dicotyledons) are typically underrepresented in phytolith samples since monocotyledons (grasses and sedges) are much more prolific producers of phytoliths. Sample GSN-16-11 is dominated by woody phytolith forms (Table 3).

**Table 3:** Absolute phytolith counts in numbers per gram of sediment for Zaraa Uul Locus 1 and Locus 2.

Sample	GSN-16-6	GSN-16-7	GSN-16-8	GSN-16-9	GSN-16-10	GSN-16-11	GSN-16-12	GSN-16-16	GSN-16-17	GSN-16-18
Context	L2	L2	L2	L2	L2	L2	L2	L1	L1	L1
**SINGLE-CELL**	n/gm	n/gm	n/gm	n/gm	n/gm	n/gm	n/gm	n/gm	n/gm	n/gm
**Long (Smooth)**	8179	8086	8160	9446	8547	270	12288	16756	16394	23616
**Long (Sinuate)**	1037	554	480	230	93	0	960	524	1108	1440
**Long (Echinate)**	3226	2437	3720	2650	2137	60	2880	3927	3323	4320
**Long (Dendritic)**	346	332	840	115	0	0	576	0	0	0
**Long (Rods)**	1152	1662	1320	1613	836	30	1920	6807	4431	7200
**Papillae:**	0	0	0	115	0	0	960	0	0	0
**Hairs:**	1728	332	120	576	650	0	384	1047	3102	2304
**Bottle-shape Hairs**	1267	2215	2400	1382	836	0	4032	2095	2880	4896
**Trichomes:**	4954	3766	3720	4032	3809	60	4800	7331	5760	8928
**Bulliform:**	1498	1329	2160	1152	2230	90	3264	5236	4431	8064
**Fan-shaped Bulliform**	115	443	840	461	1579	90	192	1833	1329	2304
***Phragmites* Bulliform**	0	0	0	230	0	0	0	0	0	0
**Crenates:**	922	443	240	461	0	0	1152	524	443	576
**Bilobes:**	806	997	840	691	279	0	1728	3665	1551	2592
**Cross-Shape**	0	0	0	346	0	0	384	262	443	288
**Rondels**	1036	7200	9240	8525	6875	30	19968	20422	20382	27360
	8									
**Stipa-Type Rondel**	346	6757	1296	9331	5203	0	17664	11520	11742	7776
			0							
**Saddles:**	1498	4985	1440	806	372	0	2880	2095	443	2016
**Cones:**	3802	997	2160	1037	1394	0	960	4713	3545	7776
**Flat Tower:**	115	111	0	0	0	0	192	0	0	0
**Smooth Spheroid**	0	0	0	115	0	0	0	0	0	288
**Elongate**	0	0	0	0	0	30	192	524	0	0
**Tracheids**	0	111	0	0	0	0	0	0	0	0
**Succulent**	0	0	120	0	0	0	0	0	0	0
**Pyramid-Shape Blocks**	0	0	0	0	0	0	0	785	886	0
**Platey**	691	332	600	230	1301	2340	576	524	665	864
**Sheet**	0	0	0	0	372	0	0	0	0	0
**Single Polyhedron**	0	0	0	0	93	0	0	0	0	0
**Verrucate**	0	0	240	0	93	60	192	0	0	0
**MULTI-CELL**	n/gm	n/gm	n/gm	n/gm	n/gm	n/gm	n/gm	n/gm	n/gm	n/gm
**Leaf/Stem: Psilate**	0	240	540	300	360	210	384	0	420	480
**Leaf/Stem: Echinate**	0	0	0	0	60	0	192	0	0	0
**Leaf/Stem: Sinuate**	60	0	0	0	0	0	0	0	0	0
**Wild Grass Husk:**	0	0	0	0	0	0	0	180	60	60
**Panicoid**	0	60	60	60	0	0	0	0	60	0
**Cyperaceae**	120	240	0	0	0	0	0	360	60	60
***Stipa* leaf/stem**	0	0	240	0	180	30	192	0	60	0
**Polyhedron**	120	0	0	300	0	840	2496	240	180	300
**Diatoms**	0	0	0	0	0	0	0	120	0	0

Trends in these samples suggest several interpretations relevant to reconstructing microenvironments at the site. First, the dominant grass genus represented is bunch grass, *Stipa* sp. (c.f. *Stipa capillata*) which is typical of the natural undegraded steppe vegetation found widely across Central Asia. The phytoliths from this genus occur in both Locus 1 and Locus 2 samples (Fig 10C). The phytolith record from Zaraa Uul also contains smaller but significant numbers of phytoliths from reed-grasses and sedges (Fig 10A and 10B). These monocotyledons typically grow in moist or wet sediments on the banks of marshes, on the bars of streams, or other microenvironments with high water tables. They are consistent with findings from the geoarchaeological field investigations which show that there were higher water tables, fresh-water marshes and active alluvial activity during the Early-Middle Holocene Period near the site [24]. Both the presence of multi-cell grass husky phytoliths (Fig 10D) and abundant concentrations (n/gm) of reed and sedge phytoliths in all three Locus 1 samples (Table 3, Fig 10A and 10B) suggest moister microenvironments being exploited during the site occupation. Dates on bone collagen and ceramic sherds from Locus 1 span 6276–1717 cal BP (Table 2). Phytolith samples were taken at 35–42 cm below datum, while dated artifacts were recovered at depths of 70–86 cm below datum. Dates are in reverse chronological order (probably due to slope wash) which indicates the phytoliths likely belong to occupations during the earliest phase of Holocene climatic amelioration. During this time, the inhabitants of Locus 1 would have been transporting vegetation from the nearby marshes to use for baskets, bedding, and/or building shelters.

### Fauna

All mapped and screened fauna were analyzed and nearly half of all bone fragments (41.7%) were identifiable to family, order, genera, species, or body size class. Excavations at Otson Tsokhio were limited in scope (2 x 2 m unit in 2018). Preservation was excellent for all assemblages.

All classes of elements (crania, vertebrae, legs, and podials) were represented in all assemblages for both large and small species. There was some variation in relative frequency of elements represented across loci and body size of prey: cranial elements were most common across the site for large ungulates (66.2% of MNI), especially at Otson Tsokhio (79.1% of MNI); large ungulate vertebral elements were most common at Locus 1 (52.9% compared to 12.2% for entire assemblage); and frequencies of both medium and small ungulate feet were higher at Locus 3. This variation is likely due less to transport and more to peculiarities of fragmentation/preservations (e.g., large ungulate teeth) and the use of bone for raw materials, rather than transport. Issues related to transport will be discussed in other publications.

Holocene and Pleistocene assemblages clearly related to human activity but reveal very different patterns of use and deposition. Degrees of fragmentation between sites were assessed by measuring maximum length of all bone fragments without any fresh breaks (Table 4, S2 Table). A Kruskal-Wallis rank sum test indicated significant variation in fragmentation across sites (*p* <0.001). Otson Tsokhio fauna was least heavily fragmented, which may relate to both the size/density of the bones and the degree of dietary or non-dietary processing. Spiral fractures are not present in the Otson Tsokhio assemblage and limited at Zaraa Uul (N Locus 2 = 13, N Locus 3 = 8), but there is evidence of cut marks on fauna from both Zaraa Uul (N Locus 2 = 8, N Locus 3 = 5) and Otson Tsokhio (N = 2). Carnivore gnawing is present at Zaraa Uul (N Locus 2 = 22, N Locus 3 = 14) and very rare at Otson Tsokhio (N = 2). Cut marks on a femoral head and the distal shaft of an ulna are related to carcass processing. Wide, irregular grooves associated with hacking were also found at Otson Tsokhio on both the spine of the rhinoceros scapula and the giant elk metatarsal. Primary processing (butchery) is therefore the main type represented at Otson Tsokhio.

**Table 4:** Summary of bone fragment measurements (mm), as recorded in S2 Table.

	ZU, Locus 2	ZU, Locus 3	Otson Tsokhio	Zuun Shovkh
**Min.**	1	1	8	10
**Max.**	99	143	118	20
**Mean**	14.3	18.2	24.4	13.9
**Sd**	12.2	12.5	15.7	3.4

Bone at Zaraa Uul shows greater evidence, not only of carnivore gnawing, but also secondary processing (post-butchery bone modification): long bones from Locus 2 are often split along the long axis (N = 107) and percussive flaking is present in both loci 2 and 3 (N = 4, N = 20, respectively). Although no hearths or ash pits were found, burning was present across Locus 1 (N = 8), Locus2 (N = 101), and Locus 3 (N = 53), including burning on hare (*Lepus tolai*) bone from Locus 2 (N = 2) and Locus 3 (N = 1). Calcination was present on only two faunal specimens, one each from Locus 2 and Locus 3, suggesting that burning was incidental to cooking rather than their use fuel. Burning was not present on bone from Palaeolithic assemblages. Taphonomic variation between Zaraa Uul and Otson Tsokhio assemblages may be related to the nature of these deposits (e.g., butchering versus cooking and manufacturing debris). Rodent gnawing is rare (N = 3 for Locus 3 and N = 1 for Otson Tsokhio).

Despite clear evidence that these assemblages were created through human activity, some of the fauna included in analysis may not have been associated with hunting and subsistence. This is likely the case with the very limited human remains, and potentially much of the microfauna. Some of the microfauna included may be intrusive, however, it can be difficult to visually determine as taphonomic processes will variously affect bones of different density and microstructure (see above). The emphasis on small to micro-sized fauna at Zuun Shovkh suggests that the faunal deposit may not be anthropogenic and etching on many of the bones indicate they may have been deposited as predator pellets or feces. This sample was therefore not included in our consideration of dietary changes.

**Fig 11:**
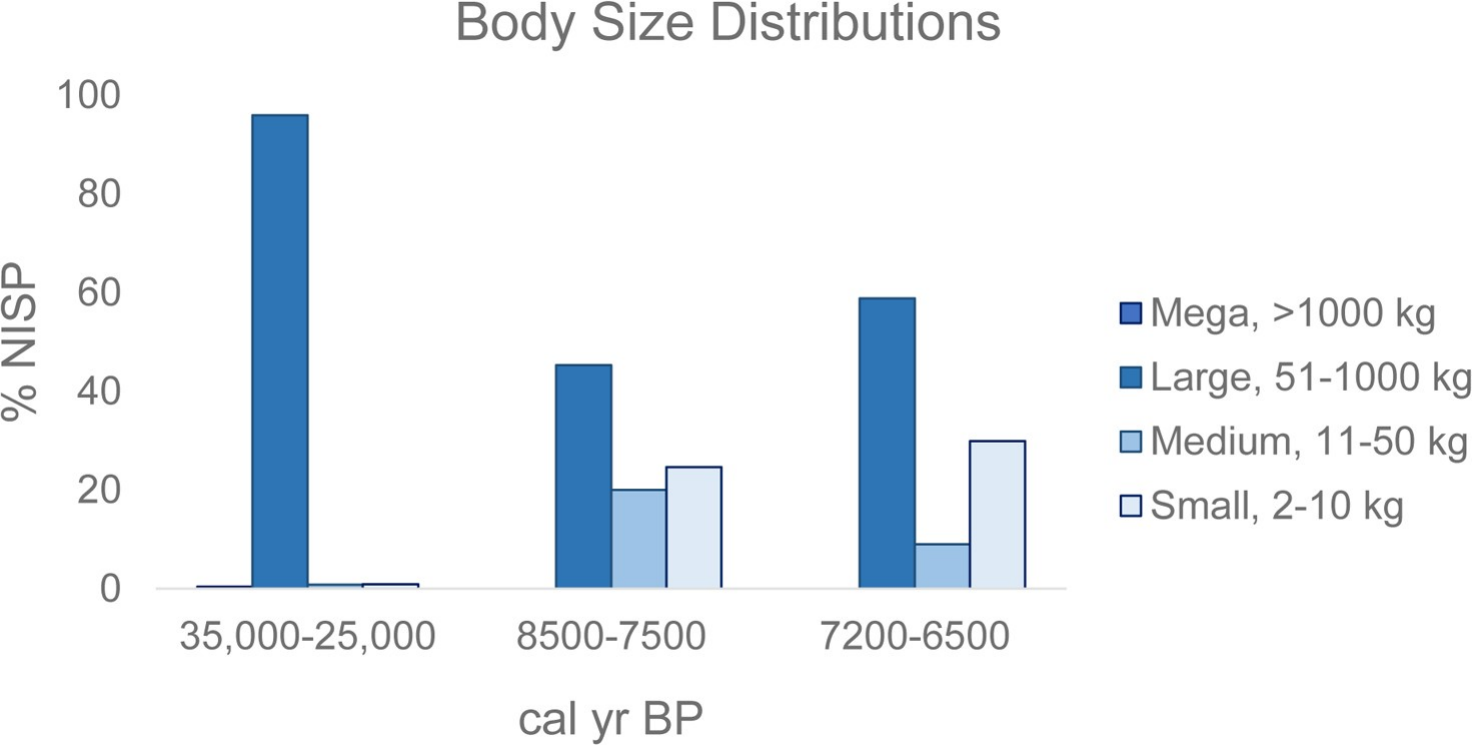
Relative frequencies of fauna across time periods according to body-size.

The majority of fauna, however, were certainly deposited as a result of human activity and show long-term consistency in favoured prey types, but notable variation in the types of complementary species exploited over time. Large-bodied fauna dominate the Late Pleistocene Otson Tsokhio assemblage at 95.9%, with medium- and small-bodied fauna comprising 0.8% and 0.9%, respectively. Ungulates comprise 98.2% of the identified fauna of Otson Tsokhio, with little evidence of avian species (0.5%) or carnivores (1.4%). Fauna from Zaraa Uul show a primary reliance on ungulates (Locus 2 = 91%, Locus 3 = 92%) with a statistically significant (*X*^2^ = 11.81, *df* = 1, *p* < 0.01) increase in both avian (Locus 2 = 4%, Locus 3 = 6%) and carnivore (Locus 2 = 5%, Locus 3 = 2.5%) species compared to the Pleistocene layers. There is a statistically significant (*X*^2^ = 468.1, *df* = 3, *p* < 0.01) shift across all body sizes of prey between 8500–7500 cal BP (Locus 2) and 7200–6500 cal BP (Locus 3) components, with an increase in large-bodied (45.5% to 58.8%) prey and corresponding decline in medium-bodied (20.0% to 9.0%), as well as small- and micro-bodied, prey (Table 5, Fig 11). Phytolith data from Locus 3 would help determine whether this pattern is of a seasonal nature or related to a long-term trend. Relative frequencies of microfauna (< 2 kg) remain fairly stable over time, but the presence of burrowing rodents and potential carnivore prey caches may confuse our understanding of the extent to which such fauna were taken as prey.

**Table 5:** Distribution of prey by body-size across all site periods, including all fauna identified to body size and/or order.

Body Size	Zuun Shovkh	Otson Tsokhio	Locus 2	Locus 3	Locus 1	Total
	N =	N =	N =	N =	N =	N =
	(% of NISP)	(% of NISP)	(% of NISP)	(% of NISP)	(% of NISP)	(% of NISP)
**Mega**	0	4	0	0	0	**4**
**(>1000 kg)**		(0.4)				**(0.1)**
**Large**	2	1086	432	620	29	**2169**
**(50 to 1000 kg)**	(8.3)	(95.9)	(45.5)	(58.8)	(87.9)	**(67.9)**
**Medium**	0	9	190	95	2	**296**
**(10 to 50 kg)**		(0.8)	(20.0)	(9.0)	(6.1)	**(9.3)**
**Small**	13	10	296	316	1	**636**
**(2 to 10 kg)**	(54.2)	(0.9)	(31.0)	(30.0)	(3.0)	**(19.9)**
**Micro**	9	23	31	23	1	**87**
**(< 2 kg)**	(37.5)	(2.0)	(3.3)	(2.2)	(3.0)	**(2.7)**
**Total NISP**	**24**	**1132**	**949**	**1054**	**33**	**3192**
	**(55.8)**	**(85.3)**	**(48.3)**	**(53.4)**	**(19.2)**	**(58.2)**
**Total NUSP**	19	195	1016	920	139	**2289**
	(44.2)	(14.7)	(51.6)	(46.6)	(80.8)	**(41.7)**
**Total Specimens**	**43**	**1327**	**1965**	**1974**	**172**	**5481**

NISP = Number of Identified Specimens, NUSP = Number of Unidentified Specimens.

Overall, the numbers suggest a continued preference for large-bodied prey, with distinct variation in relative frequencies over time. A nearly exclusive preference for large-bodied prey is evidenced in the Pleistocene component while large-bodied prey represent closer to 50% of the Holocene assemblage. We also note a shifting emphasis from medium- to large-bodied prey between the early to later phases of Holocene occupation. This is surprising as we would expect large-bodied prey to decline under the pressure of prolonged human predation; however, residential mobility at Zaraa Uul may not ever have been low enough, or population density high enough, to depress prey populations. The shift might rather be related to seasonal differences in site use over time or environmental change through the millennia, or could be a symptom of unknown sampling bias. As such, future analysis should focus on determining whether there was an actual decline in medium-bodied prey and whether this was balanced by a greater emphasis on small prey.

Otson Tsokhio fauna includes *Camelus ferus* [camel], *Coelodonta antiquitatis* [woolly rhinoceros], *Equus ferus* [wild horse], *Equus hemionus* [wild ass/khulan], Bovinae [aurochs or bison], *Megaloceros* sp. [giant elk], *Ovis ammon* [argali sheep], Caprinae [argali sheep or ibex], Antilopinae, Hyaenidae [hyaena], and *Canis* sp. [dog]. The faunal assemblage from Zaraa Uul includes a greater diversity of species types, including many that are expected in the arid steppe environment (*Equus* spp., *Bos primigenius*, Antilopinae [*Saiga tatarica*, *Gazella subgutturosa*, and/or *Procapra gutturosa*], *Vulpes* spp. [red fox and/or corsac fox], c.f. *Lepus tolai*, *Ochotona* spp. [pika], and Accipitridae [bird of prey]) as well as several other species that are prefer forested or shrubby habitats and are regionally extinct or rare (Cervidae [deer], *Sus scrofa* [wild boar], *Panthera tigris* [tiger], *Nyctereutes procyonoides* [raccoon dog], medium-sized Mustelidae [c.f. *Meles leucurus* or badger], and *Phasianus colchicus* [pheasant]) (Table 6). The *Panthera tigris* (tiger) bone dates to 6640–6494 cal BP (5765 ± 25). This find is ecologically consistent with the presence of key prey species such as deer and boar that were also being hunted by humans. The bone is from an animal’s paw (left 3^rd^ metatarsal) and as such may be a remnant of skin used for clothing or furnishing. Equids represent the primary category of identifiable large-bodied prey from the Upper Palaeolithic through the early Oasis 2 phase of occupation, whereas identified specimens in the later phase of Oasis 2 are dominated by bovines (Table 6). Middle Holocene layers contain *Equus hemionus* (khulan or wild ass), where equid species are identifiable to species (based on size), while both *Equus hemionus* and *Equus ferus* (wild horse) are present in Pleistocene layers.

**Table 6:** NISP distribution of all fauna identifiable to order. Zuun shovkh (ZSh) and Otson Tsokhio (OTs) date between 35,000–25,000 cal BP. ZU Locus 2 (ZU2.T2/3) dates at 8500–7500 cal BP. ZU Locus 3 (ZU3.T1) dates at 7500–6500 cal BP. ZU Locus 1 (ZU2.T1) is a mixed deposit with dates spanning 3495–1717 cal BP. Fauna classified only by body size are not included.

Order	Family	Species/Size Class	ZSh	OTs	ZU, L2	ZU, L3	ZU, L1
**Primates**	**Hominidae**	***Homo sapiens sapiens***			1	1	
**Rodentia**	**Unknown**			6 (1*)	10	12 (1*)	1
	**Cricetidae**		1*	2			
	**Muridae**					1	
	**Dipodidae**					1	
	**Sciuridae**	***Marmota sibirica***			63^a^		
**Lagomorpha**	**Unknown**				2		1
	**Leporidae**	***Lepus tolai***	1		33	48	
	**Ochotonidae**	***Ochotona* sp.**		1	3	1	
		***Ochotona pallasi***			3*		
**Carnivora**	**Unknown**		3	1	3	1	
	**Canidae**	**Unknown**				1	
		***Canis* sp.**		1			
		***Nyctereutes procyonoides***	1		2		
		***Vulpes* sp.**			6		
		***Vulpes corsac***				1	
	**Hyaenidae**			2			
	**Felidae**	***Panthera tigris***				1	
	**Mustelidae**	***Gulo* sp.**	1				
		***Meles leucurus***			1		
		***Martes* sp.**			1		
**Perissodactyla**	**Rhinocerotidae**	***Coelondonta antiquitatus***		1			
	**Equidae**	***Equus* sp.**		11	35		3
		***Equus ferus***		4			
		***Equus hemionus***		5		3	
**Artiodactyla**	**Camelidae**	***Camelus ferus***		1			
	**Bovidae**	**Unknown Bovidae**			4	1	2
		**Bovinae** (*Bos primigenius* or *Bison* sp.)		2	9 (3 *Bos*)	13 (2 *Bos*)	4 (3 *Bos*)
		**Caprinae** (*Ovis ammon* or *Capra sibirica*			5	1	
		***Ovis ammon*** (large)		1			
		**Antilopinae** (*Saiga tatarica*, *Gazella subgutturosa* or *Procapra gutturosa*)		1	8	6	
		**Medium Bovidae** (Caprinae/large Antilopinae)			12	1	
		**Small Bovidae** (Antilopinae/ small Caprinae)			5	2	
	**Cervidae**	***Megaloceros* sp**.		2			
		**Large Cervidae** (*Alces alces cameloides*, or large *Cervus elaphus*)			2	2	
		**Medium Cervidae** (small *C*. *elaphus*, *C*. *nippon* or *E*. *davidianus*)		1	1	3	
		**Small Cervidae** (*Capreolus pygargus*)			3	1	
	**Suidae**	***Sus scrofa***			1	5	
**Falconiformes**	**Accipitridae**				1		
**Galliformes**	**Phasianidae**	***Perdix dauuricae***				1	
		***Phasianus colchicus***			1	2	
**Columbiformes**		***Columbia* sp.**			1		
**Passeriformes**						1	
**Anura**			1		1		
**TOTAL**				18	214	107	

^a^Only two individuals are represented for *Marmota sibirica*.

*Indicates that specimen is likely intrusive.

Shell was not considered in faunal analysis as we consider the primary function to be raw material. A bead on, and several larger fragments of, ostrich [*Struthio asiaticus*/*anderssoni*] eggshell was recovered at Otson Tsokhio and both of the two fragments we dated pre-date the fauna by several thousand years. Mother-of-pearl (*nacre*) was recovered in both Locus 2 and 3 (Fig 2), found in both fragmentary clusters and in one section of Locus 3 as two flower-shaped beads (Fig 2). These derived from a local species of freshwater mussel (*Uno* sp.). Such artifacts have been found in burial contexts to the north, in the far eastern steppe region of Dornod province. *Uno* spp. may have inhabited the basin at Zaraa Uul or been imported from the north, where the shells are better represented archaeologically at sites like Tamsagbulag and Hag (Inner Mongolia) [54].

## Discussion

Despite challenges in identifying archaeological fauna (e.g., fragmentation and limited comparative collections) to species and the resulting impact on high resolution interpretations of the dietary composition among ancient animal communities, there is a demonstrated shift in species composition over time: 1) Late Pleistocene faunal communities were characterized by grassland species including large-bodied mixed-feeders (e.g., woolly rhinoceros, giant elk, camel) and large-bodied grazers (e.g., equids); and 2) mid-Holocene communities represent a mix of grassland or forest-steppe adapted large-bodied grazers (e.g., equids, wild cattle/aurochs) and medium-bodied mixed-feeders (e.g., gazelle), complemented by forest-adapted large- to small-bodied browsers (i.e., cervids). Pleistocene landscapes in the north, which were forested in the Holocene, are likewise thought to have been dominated by expansive mosaic grasslands with widely dispersed arboreal and shrub vegetation which supported a diverse range of generalized herbivores [55]. From the Bronze Age to modern times, grassland-adapted large- and medium-bodied grazers (e.g., domesticated horses, cattle, and sheep) dominate alongside medium- to small-bodied mixed-feeders (e.g., domesticated goats); browsers are rare.

It could be argued that variation in these assemblages resulted entirely from changes in dietary preference rather than ecological change; however, there are other regional indicators of massive ecological shifts, including large-scale extinctions of large herbivores. The post-LGM extinction of giant elk and ostrich [56, 57] are notable examples. Both species require high calcium inputs to support antler development and egg laying, respectively. During the Pleistocene, the availability of saline lakes, salt tolerant vegetation, and exposure of associated mineral-rich soils and salt tolerant vegetation during the Pleistocene would have advantaged such adaptations. In contrast, post-glacial aeolian and alluvial deposition around former saline lakebeds would have altered the availability of these soils and influenced similar vegetative changes to those described by Rosen et al. for the early Bronze Age [33].

Diachronic changes in the distribution and diversity of ungulate species underscore the unique nature of Early-Middle Holocene environments compared to those of the Pleistocene. While the persistence and continuous use of certain genera of steppe fauna (e.g., *Equus* spp., *Bos* spp., *Ovis* spp.) highlights the importance of grazing ungulates within steppe environments across time, including the importance of these ungulate species for human survival in diverse steppe environments, the extinction of large- and medium-bodied mixed feeders such as woolly rhinoceros and giant elk points to large-scale ecological shifts. Mixed feeders have flexible browsing/grazing strategies, but avoid cellulose-rich grasses in favour of quality diets that meet their high nutritional needs [58]. An increase in cervid diversity at Zaraa Uul corresponds to the expectations outlined in Guthrie’s [55] stripes and mosaics model. Following this model, Janz [10] has argued that ecological fragmentation was the major contributing factor to global shifts in human foraging. This model posits that arboreal, shrub and grassland vegetation were more homogeneously distributed during most of the Pleistocene, but that Holocene environments were characterized by increasing segregation into closed forests and open grasslands. This would have made sufficient access to high quality forage more challenging for very large-bodied ungulates adapted to open-canopy environments but reliant on browse. The presence of a variety of large to small browsing species during the Early-Middle Holocene supports the model.

The appearance of specialized browsers also highlights both widespread evidence for enhanced humidity and increases in arboreal vegetation across the Gobi Desert during the Early-Middle Holocene [10, 11, 22, 53]. Shifting archaeological representation of equines relative to bovines is further compelling with respect to palaeoecology as bovines are better adapted to forest-steppe environments; this shift might likewise highlight the culmination of arboreal vegetative growth. Another explanation for *Bos primigenius* becoming increasingly important over time is a culturally-mediated shift towards specialized cattle exploitation, the possibility of which would imply the spread of specialized cattle exploitation represented at northern steppe sites like Tamsagbulag [23, 54]. Bovines are not, however, as intensively exploited at Zaraa Uul as at Tamsagbulag.

The decline of these species during the late Holocene reflects the trend towards deforestation, greater aridity, and the increasing dominance of domesticated mixed-feeders. The modern anthropogenic landscape is the product of at least three thousand years of pastoralist activity coupled with Late Holocene aridity. Trees and substantial shrubs are rare and the largely open plains are dominated by herbaceous vegetation like *Allium polyrhizum* (wild onion) with patches of tough *Achnatherum splendens* (derse) in heavier soils. Modern steppe fauna are primarily domesticated species who are specialized grazers, including *Equus ferus caballus*, *Bos taurus*, *Ovis aries* [58], with the few successful mixed-feeder species represented by the small-bodied domesticate *Capra aegagrus hircus* alongside wild *Procapra gutturosa* (Mongolian gazelle or *dzeren*) and wild *Ovis ammon darwini* (argali sheep). Camel (*Camelus ferus* and *Camelus bactrianus*) is not suited to the modern desert-steppe environment of Zaraa Uul; wild species are locally extinct and local herders told use that camel husbandry was challenging in this region.

Ecological changes as reflected in herbivore communities highlight the relationship between changes in human foraging and intensified wetland use, particularly the importance of species diversity. The presence of pheasant, raccoon dog, deer, boar, and tiger also suggest thicker brush, shrub and arboreal vegetation and substantial wetlands, the latter further supported by the presence of sedge phytoliths. Based on the faunal, geomorphological, and phytolith data, we hypothesize that the surrounding steppe was much less arid, while the basin contained marsh, freshwater ponds, and possibly lake deposits in the deepest sections of the basin. A substantial spread in arboreal and shrub vegetation has been hypothesized [10, 11] and we suggest that trees and shrubs would have lined flowing rivers and drainage channels. Usewear analysis of chipped stone adzes from Zaraa Uul and other Gobi Desert sites supports this idea: these tools, first introduced during Oasis 2, were primarily used–and likely designed–for woodworking [59]. Phytoliths from woody shrubs or trees and the intensive use of adzes at Zaraa Uul could be connected to woodworking in Locus 2. The presence of arboreal vegetation is further supported by pollen data from the region around Delgerkhaan Uul [53]. Enhanced vegetation cover combined with the type of freshwater ponding described herein would have been likely to attract ample waterfowl and small aquatic mammals, some of which were exploited by inhabitants at Zaraa Uul. While fish are known to be abundant in stable rivers and lakes even in the more arid conditions of the 20^th^ century [60, 61], there is no evidence that they were being exploited locally. In short, post-glacial forestation and wetland expansion significantly altered species composition from open steppe to an arid forest-steppe-wetland mosaic wherein hunter-gatherers could exploit a range of small and large terrestrial prey and semi-aquatic species.

Foraging strategies shifted in response to these ecological changes. Wetlands nurture and attract a greater diversity of plants and animals relative to other environments, particularly in arid settings where arboreal growth is heavily restricted. Increased evidence for plant processing during Oasis 2 (Early-Middle Holocene) is probably closely connected to the appeal of freshwater marshes as base camps; access to starchy plants and prey with restricted territorial ranges (e.g., deer, hare, pheasant) would have provided stable foraging opportunities while large game could be hunted in the surrounding steppes. The range of species present and the numerous adzes on-site suggest that forestation of uplands and river drainages was well-developed, and this would have offered additional resources. It is likely that oasis environments enabled settlement of arid regions in at levels that were impossible during other periods. Evidence of an intensive wetland-plant-based diet among Saharan hunter-gatherers during a similar wet phase in northern Africa [7] offers a potential comparison in light of innovations in plant processing technology that appear in the Gobi Desert during Oasis 2.

Organizational changes enacted among wetland-based hunter-gatherers during the Early-Middle Holocene represent a shift in material culture and subsistence that spread across Northeast Asia during this period, including the widespread adoption of pottery, the incorporation of milling technology, the use of polished stone tools, and the trend towards heightened sedentism across the Mongolian Plateau, northern China, and much of the rest of Northeast Asia [10, 54, 62–64]. The ecological relationship between greater diet breadth, more intensive use of particular sites, and climatic amelioration further supports the emerging realization that increasing sedentism and enhanced diet breadth were closely intertwined with Holocene ecological changes. These ecological changes and human responses were repeated in many regions and should be recognized as transformative for human societies at a global scale [10, 65].

## Conclusion

Intensive occupation around Zaraa Uul is tied to two climatic phases: Early Upper Palaeolithic occupations at Otson Tsokhio and Zuun Shovkh, during the last Pleistocene MIS 3 interglacial; and the Oasis 2 occupation during the Holocene Climatic Optimum. This study confirms the development of a distinct Early-Middle Holocene ecosystem in the Gobi Desert, correlating with unprecedented socio-economic changes that include reduced residential mobility–a pattern repeated across Northeast Asia at this time. This manifested as singularly high levels of sedentism on the eastern edge of the Mongolian Plateau [54]. Response to ecological change in the Gobi Desert was characterized by the development of logistical foraging bases from which hunter-gatherers could persistently exploit a diversity of ecozones [10]. The phytolith and faunal records from Zaraa Uul reinforce the hypothesis that wetlands were a primary focus because of the denser and more diverse range of plant and animal species that they supported, with wetland-based camps allowing access to range of small, medium and large prey associated with arid steppe, arboreal, and wetland habitats [10, 22].

The progressive drying of freshwater marshes after 6400 cal BP may have reduced the productivity of this environment for the type of substantive human occupation demonstrated between 8500–6500 cal BP. A decreased emphasis on medium and small-bodied prey after 7200 cal BP is noteworthy but we are unable to offer an explanation. The locality was apparently not used regularly after 6500 cal BP until the widespread adoption of herding. Additional environmental and archaeological data are needed from the region around Zaraa Uul in order to understand the more general character of land-use strategies, particularly at ~6000–3500 cal BP during the local archaeological hiatus. This hiatus suggests a relationship between increasing aridity and disuse or changing use of the site. Regular site use recommences during the early Bronze Age is recognizable by the many concentrations of burial cairns scattered along the south-facing ridges of the Zaraa Uul range [22]. Various phases of later low density habitation are recorded by radiocarbon dates on ceramics from Locus 1 (ZU2.T1) and bone from Locus 3 (ZU3.T1). The dates correspond with Bronze and Iron Age burial activity.

The adoption of pastoralism probably stimulated the reoccupation of Zaraa Uul during the Bronze Age. Herding is highly adaptable to arid regions as its practioners are able to produce potable fluids and a reliable source of protein when both are environmentally scarce. If Zaraa Uul was unamenable to the foraging lifestyle after ~6500 cal BP, the spread of pastoralism may have served to reopen such habitats to human use.

Zooarchaeological data highlights substantive changes in the nature of faunal communities: 1) local extinctions of numerous large-bodied herbivores and carnivores (e.g., *Struthio asiaticus/anderssoni* [ostrich], *Coelodonta antiquitatis* [woolly rhinoceros], *Megaloceros* sp. [giant elk]) and Hyaenids (e.g., *Crocuta crocuta spelaea*) prior to or during the early Holocene; 2) increased diversity of smaller-bodied and more specialized herbivores during the Middle Holocene Climatic Optimum; and 3) late Holocene aridification and the rise of domesticated herd animals, which corresponds with the local retreat of species such as cervids, wild boar, and various small-bodied species (e.g., pheasant, badger, and raccoon dog). Divergent faunal communities during Pleistocene and Holocene wet phases underscores epochal changes in global ecology. Locally, in the Gobi Desert, the Holocene is characterized by the late Pleistocene/early Holocene evaporation and infilling of deep saline lake basins, resulting in the widespread development of freshwater marshes, enhanced vegetative biomass, and increasing species diversity during the Holocene.

Late Pleistocene and Early-Middle Holocene wet phases were demonstrably amenable to human occupation, but variation in land-use [10, 11] reflects the unique and highly divergent ecologies associated with each of these wet phases, as reflected in hydrology, vegetation, and fauna. Differing hydrological regimes and their influence on processes such as alluvial deposition, characterized herein by a change from saline lakes to freshwater marshes, were deeply connected to ecological conditions moderating vegetative and faunal communities. The unique diversity of Holocene fauna highlights the substantive nature of hydrological and vegetative changes, which were interconnected with large-scale megafaunal extinctions [55, 57]. Human response to these ecological changes in arid Northeast Asia is demonstrated by the intensive use of Zaraa Uul and other wetland oases during the Holocene Climatic Optimum. Our results highlight the importance of archaeological data not only for understanding the relationship between social and environmental change, but also for gaining a more dynamic understanding of ecological relationships resulting from specific climatic events.

## Supporting information

S1 Table List of mammals likely to have been present in region during Pleistocene and Holocene epochs according to body size and taxonomy. Includes range of body weights, body sizes, and ecological niche [34].
(XLSX)

S2 Table Faunal remains used in analysis and all characteristics recorded for each element (“faunal remains”), including key for codes (“key”). All measurements for Table 4 are included in the sheet “Fragment Measurements”.
(XLSX)

## References

[1] Nicholas GP. Wetlands and hunter-gatherers: a global perspective. Curr Anthropol. 1998; 39(5): 720–731.

[2] Gamble C. The Mesolithic sandwich: ecological approaches and the archaeological record of the early post glacial. In: Zvelebil M, editor. Hunters in Transition: Mesolithic Societies of Temperate Eurasia and their Transition to Farming. Cambridge: Cambridge University Press; 1986. pp. 33–42.

[3] Hillman GC. Late Paleolithic plant foods from Wadi Kubbaniya in Upper Egypt: dietary diversity, infant weaning, and seasonality in a riverine environment. In: Harris DR, Hillman GC, editors. Foraging and farming: the evolution of plant exploitation. London: Unwin Hyman; 1989. pp. 207–239.

[4] Fuller DQ, Qin L. Declining oaks, increasing artistry, and cultivating rice: the emergence of farming in the Lower Yangtze region. Environ Archaeol. 2010; 15(2): 139–159.

[5] Smith BD. The cultural context of plant domestication in eastern North America. Curr Anthropol. 2011; 52: S471–S484.

[6] Elston RG, Zeanah DW, Codding BF. Living outside the box: an updated perspective on diet breadth and sexual division of labor in the Prearchaic Great Basin. Quatern Int. 2014; 352(1): 200–211.

[7] Dunne J, Mercuri AM, Evershed RP, Bruni S, di Lernia S. Earliest direct evidence of plant processing in prehistoric Saharan pottery. Nat Plants. 2016; 3: 16194. 10.1038/nplants.2016.194 27991880

[8] Capriles JM, Lombardo U, Maley B, Zuna C, Veit H, Kennett D J. Persistent Early to Middle Holocene tropical foraging in southwestern Amazonia. Sci Adv. 2019; 24: eaav5449. 10.1126/sciadv.aav5449 31032413 PMC6482008

[9] Ramsey MN, Rosen AM. Wedded to wetlands: exploring late Pleistocene plant-use in the eastern Levant. Quatern Int. 2016; 396: 5–19.

[10] Janz L. Fragmented landscapes and economies of abundance: the Broad Spectrum Revolution in arid East Asia. Curr Anthropol. 2016; 57(5): 537–564.

[11] Janz, L. Chronology of Post-Glacial Settlement in the Gobi Desert and the Neolithization of Arid Mongolia and China. PhD thesis, University of Arizona. 2012. Available from: https://repository.arizona.edu/handle/10150/223342

[12] Maringer J. Contribution to the Prehistory of Mongolia. Reports from the Scientific Expedition to the North-western Provinces of China under the Leadership of Sven Hedin, Sino-Swedish Expedition Publication, Publication 34. Stockholm: Statens Etnografiska Museum, Stockholm; 1950.

[13] Fairservis WA. The Archaeology of the Southern Gobi, Mongolia. Durham, NC: Carolina Academic Press; 1993.

[14] Berkey CP, Nelson NC. Geology and prehistoric archaeology of the Gobi Desert. Am Mus Novit. 1926; 222: 9–16.

[15] Nelson NC. The dune dwellers of the Gobi. Nat Hist. 1926a; 26: 246–251.

[16] Nelson NC. Notes on the archaeology of the Gobi Desert. Am Anthropol. 1926b; 28: 305–308.

[17] Gábori M. Beiträge zur typologie und verbreitung der Shabarakh-Kultur. Acta Archaeol-Bud. 1962; XIV(3/4): 59–174.

[18] Okladnikov AP. Новые данные по древнейшей истории внутренней Монголии [New data on the ancient history of Inner Mongolia]. Vestn Drevnej Istor (Moskva). 1951; 4: 162–174.

[19] Okladnikov AP. Новое в изучении древнейших культур Монголии (по работам 1960 г.) [New in studies on the ancient culture of Mongolia–work to 1960]. Sov etnografiya. 1962; 1: 83–90.

[20] Kozłowski JK. Research on the Stone Age in South Mongolia in 1968. Archaeol Pol. 1972; 13: 231–261.

[21] Perlee Kh, Ser-Odjav N. Дорноговиос эртний хүний ул мөр олдлоо (Found traces of an ancient man in Dornogov’). Sci Technol-Mong. 1957: 5–6.

[22] Janz L, Odsuren D, Bukhchuluun D. Transitions in palaeoecology and technology: hunter-gatherers and early herders in the Gobi Desert. J World Prehist. 2017; 30(1): 1–55. 10.1007/s10963-016-9100-5

[23] Odsuren D., Bukhchuluun D., Janz L. Дорнод Монголд хийсэн шинэ чулуун зэвсгийн үеийн судалгааны зарим үр дүн (Preliminary results of Neolithic research conducted in eastern Mongolia). Stud Archaeol. 2015; 35: 72–96.

[24] Rosen A. M., Odsuren D., Bukhchuluun D., Janz L. Holocene Desertification and Human Resilience in the Eastern Gobi Desert. For submission to PLoS ONE.

[25] Odsuren D, Bukhchuluun D, Janz L, Rosen A. “Говь, хээрийн бүсийн неолитын судалгаа” төслийн 2016 оны хээрийн шинжилгээний ажлын үр дүн (“Gobi-Steppe Neolithic Project,” results of 2016 expedition). Mong Archaeol. 2017; 2016: 22–28.

[26] Tumen D, Khatanbaatar D, Erdene M. Bronze Age graves in the Delgerkhaan Mountain area of eastern Mongolia. Asian Archaeol. 2014; 2: 40–49.

[27] Honeychurch W. Inner Asia and the Spatial Politics of Empire: Archaeology, Mobility, and Culture Contact. New York: Springer Publications; 2015.

[28] Wright J, Ganbaatar G, Honeychurch W, Byambatseren B, Rosen AM. The earliest Bronze Age culture of the South-eastern Gobi Desert, Mongolia. Antiquity. 2019; 93(368): 393–411.

[29] Beaumont W, Beverly R, Southon J, Taylor RE. Bone preparation at the KCCAMS laboratory. Nucl Instrum Meth B. 2010; 268(7–8): 906–909.

[30] Janz L, Feathers J, Burr GS. Dating surface assemblages using pottery and eggshell: assessing radiocarbon and luminescence techniques in Northeast Asia. J Archaeol Sci. 2015; 57: 119–129.

[31] Crann CA, Murseli S, St-Jean G, Zhao X, Clark ID, Kieser WE. First status report on radiocarbon sample preparation at the A.E. Lalonde AMS Laboratory (Ottawa, Canada). Radiocarbon. 2016; 59(3): 695–704.

[32] Bronk Ramsey C. Bayesian analysis of radiocarbon dates. Radiocarbon. 2009; 51: 337–360.

[33] Rosen AM, Hart TC, Farquhar J, Schneider JS, Tserendagva Ya. Holocene vegetation cycles, land-use, and human adaptations to desertification in the Gobi Desert of Mongolia. Veg Hist Archaeobot. 2019; 28: 295–309.

[34] Batsaikhan N, Samiya R, Shar S, King SRB. A Field Guide to Mammals of Mongolia. London: Zoological Society of London; 2010.

[35] Boldbaatar Sh, Tugsbayar Sh. Photo Guide to Birds of Mongolia. 2nd Edition. Ulaanbaatar: Mongolian Foundation of Birds of Prey & TUGS, LLC; 2013.

[36] Boessneck J, Jéquier J-P, Stampfli HR. Seeburg Burgäschisee-Süd. Teil 3: Die Terreste. Bern: Verlag Stämpfli & Cie Bern; 1963.

[37] Balkwill DM, Cumbaa SL. A Guide to the Identification of Postcranial Bones of *Bos taurus* and *Bison bison*. Syllogeus No. 71. Ottawa: Canadian Museum of Nature; 1992.

[38] Lu P, Brunson K, Yuan J, Li Z. Zooarchaeological and genetic evidence for the origins of domestic cattle in ancient China. Asian Perspect. 2017; 56(1): 92–120. 10.1353/asi.2017.0003

[39] Gladyshev SA, Olsen JW, Tabarev AV, Kuzmin YV. Chronology and periodization of Upper Palaeolithic sites in Mongolia. Archaeol Ethnol Anthropol Eurasia. 2010; 38(3): 33–40.

[40] Zwyns N, Paine CH, Bolorbat T, Talamo S, Fitzsimmons KE, Angaragdulguun G, et al. The northern route for human dispersal in Central and Northeast Asia: new evidence from the site of Tolbor-16, Mongolia. Nat Sci Rep 2019; 9: 11759. 10.1038/s41598-019-47972-1 31409814 PMC6692324

[41] Batbaatar J, Gillespie AR, Fink D, Matmon A, Fujioka T. Asynchronous glaciations in arid continental climate. Quat Sci Rev. 2018; 182: 1–19.

[42] Shi Y, Yu G, Liu X, Li B, Yao T. Reconstruction of the 30–40 ka BP enhanced Indian monsoons climate based on geological records from the Tibetan Plateau. Palaeogeogr Palaeoclimatol Palaeoecol. 2001; 169: 69–83.

[43] Grunert J, Lehmkuhl F. Aeolian sedimentation in arid and semi-arid environments of western Mongolia. In: Smykatz-kloss W, Zöller L, Felix-Henningsen P, editors. Paleoecology of Quaternary Drylands. Lecture Notes in Earth Sciences, Vol. 102. New York: Springer Publications; 2004. pp. 195–218.

[44] Herzschuh U. Palaeo-moisture evolution in monsoonal Central Asia during the last 50,000 years. Quat Sci Rev. 2006; 25: 163–178.

[45] Feng Z-D, Zhai XW, Ma YZ, Huang CQ, Wang WG, Zhang HC, et al. Eolian environmental changes in the Northern Mongolian Plateau during the past ~35,000 yr. Palaeogeogr Palaeoclimatol Palaeoecol. 2007; 245: 505–517.

[46] Reimer P, Austin W, Bard E, Bayliss A, Blackwell P, Bronk Ramsey C, et al. The IntCal20 Northern Hemisphere Radiocarbon Age Calibration Curve (0–55 cal kBP). Radiocarbon 2020; 62(4): 725–757. 10.1017/RDC.2020.41

[47] Winkler MG, Wang PK. The late-Quaternary vegetation and climate change of China. In: Wright HE Jr., Kutzbach JE, Webb T III, Ruddiman WF, Street-Perrott, FA, Bartlein PJ, editors. Global Climates since the Last Glacial Maximum. Minneapolis-St. Paul: Minnesota Press; 1993. pp. 221–261.

[48] Felauer T, Schlütz F, Murad W, Mischke S, Lehmkuhl F. Late Quaternary climate and landscape evolution in arid Central Asia: a multiproxy study of lake archive Bayan Tohomin Nuur, Gobi Desert, southern Mongolia. J Asian Earth Sci 2012; 48: 125–135.

[49] Wen R, Xiao J, Fan J, Zhang S, Yamagata H. Pollen Evidence for a Mid-Holocene East Asian Summer Monsoon Maximum in Northern China. Quat Sci Rev 2017; 176: 29–35.

[50] Lee MK, Lee YI, Lim HS, Lee JI, Yoon HI. Late Pleistocene–Holocene records from Lake Ulaan, southern Mongolia: implications for East Asian palaeomonsoonal climate changes. J Quat Sci 2013; 28(4): 370–378.

[51] Holguín LR, Sternberg T. A GIS based approach to Holocene hydrology and social connectivity in the Gobi Desert, Mongolia. Archaeol Res Asia 2018; 15: 137–45.

[52] Bazarova VB, Tsydenova NV, Lyaschevskaya MS, Khenzykhenova FI, Tumen D, Erdene M. Reconstruction of Paleoenvironmental Conditions of Ancient People Habitation in the Togootyn Gol River Valley (Eastern Mongolia). Quatern Int. 2019; 503: 105–114.

[53] Jigjidsuren S, Johnson DA. Forage Plants in Mongolia. Ulaanbaatar: Admon; 2003.

[54] Zhao C, Janz L, Bukhchuluun D, Odsuren D. Neolithic pathways in East Asia: early sedentism on the Mongolian Plateau. Antiquity. 2021; 95(379): 45–64.

[55] Guthrie RD. Mosaics, allelochemics and nutrients: an ecological theory of late Pleistocene megafaunal extinctions. In: Martin PS, Klein RG, editors. Quaternary Extinctions: A Prehistoric Revolution. Tucson, AZ: University of Arizona Press; 2018. pp. 259–298. (56 to 55)

[56] Janz L, Elston RG, Burr GS. Dating Northeast Asian surface assemblages with ostrich eggshell: implications for palaeoecology and extirpation. J Archaeol Sci 2009; 36: 1982–1989. (57 to 56)

[57] Stuart AJ, Kosintsev PA, Higham TFG, Lister AM. Pleistocene to Holocene extinction dynamics in giant deer and woolly mammoth. Nature 2004; 431: 684–689. (58 to 57) 10.1038/nature02890 15470427

[58] Hofmann RR. Evolutionary steps of ecophysiological adaptation and diversification of ruminants: a comparative view of their digestive system. Oecologica. 1998; 78: 443–457. (55 to 58) 10.1007/BF00378733 28312172

[59] Evoy, A. Neolithic Resource Use and Adaptation in the Eastern Gobi Desert: Functional Analysis of Azes and Adzes. MA thesis, Trent University. 2019. Available from: https://digitalcollections.trentu.ca/islandora/search/evoy?type=dismax

[60] Nelson NC. Diary of the Central Asiatic Expedition into Mongolia, Saturday, April 18 to Wednesday, September 16. Department of Anthropology Archives, American Museum of Natural History; 1925.

[61] Hedin S. History of the Expedition in Asia 1927–1935, Part I. Reports from the Scientific Expedition to the North-Western Provinces of China under the Leadership of Dr. Sven Hedin, Publication 23. Stockholm: Statens Etnografiska Museum; 1943.

[62] Habu J. Early sedentism in East Asia: from late Palaeolithic to early agricultural societies in insular East Asia. In: Renfrew C, Bahn PG, editors. The Cambridge World Prehistory. Cambridge: Cambridge University Press; 2014. pp. 724–741.

[63] Popov AN, Tabarev AV, Mikishin YA. Neolithization and ancient landscapes in southern Primorye, Russian Far East. J World Prehist. 2014; 27: 247–261.

[64] Shelach-Lavi S, Teng M, Goldsmith Y, Wachtel I, Stevens CJ, Marder O, et al. Sedentism and plant cultivation in Northeast China emerged during affluent conditions. PLOS ONE. 2019; 14(7): E0218751. 10.1371/journal.pone.0218751 31318871 PMC6638895

[65] Project ArchaeoGLOBE. Archaeological assessment reveals Earth’s early transformation through land use. Science 2019; 365(6456): 897–902. 10.1126/science.aax1192 31467217

